# Limited population genetic variation but pronounced seascape genetic structuring in populations of the Mediterranean mussel (*Mytilus galloprovincialis*) from the eastern Adriatic Sea

**DOI:** 10.1002/ece3.9729

**Published:** 2023-01-24

**Authors:** Joanna S. Hamilton, Marina Piria, Ana Gavrilović, Mirna Mrkonjić Fuka, Lidija Svečnjak, Slađana Nikolić, Rigers Bakiu, Jonathan P. A. Gardner

**Affiliations:** ^1^ School of Biological Sciences Victoria University of Wellington Wellington New Zealand; ^2^ Department of Fisheries, Apiculture, Faculty of Agriculture, Wildlife Management and Special Zoology University of Zagreb Zagreb Croatia; ^3^ Department of Ecology and Vertebrate Zoology, Faculty of Biology and Environmental Protection University of Łódź Łódź Poland; ^4^ Department of Microbiology, Faculty of Agriculture University of Zagreb Zagreb Croatia; ^5^ Institute of Marine Biology University of Montenegro Kotor Montenegro; ^6^ Department of Aquaculture and Fisheries, Faculty of Agriculture and Environment Agricultural University of Tirana Tirana Albania; ^7^ Albanian Center for Environmental Protection and Sustainable Development Tirana Albania

**Keywords:** aquaculture, environmental variation, gene flow, genetic cline, Mediterranean Sea, Mollusca, Bivalvia, selection

## Abstract

Population genetic analysis of variation at five neutral microsatellite loci for Mediterranean mussels (*Mytilus galloprovincialis*) from 18 sites along the eastern Adriatic Sea revealed little or no spatial variation. In contrast, seascape genetics analysis revealed a pronounced locus‐specific gradient in allelic and genotypic frequencies across the study region. At a sixth locus, MGE7, the frequencies of two alleles, MGE7^243^ and MGE7^249^, were strongly associated, negatively and positively, respectively, with a single environmental variable – minimum salinity (minSAL). The frequency of the MGE7^243/243^ homozygous genotype was strongly negatively associated with minSAL, whereas the frequencies of the MGE7^246/249^ and the MGE7^249/249^ genotypes were strongly positively correlated with minSAL. Interpretation of these pronounced gradients is confounded by the fact that minSAL and another environmental variable, maximum sea surface temperature (maxSST), are highly correlated (*R* = −.911) and are therefore not necessarily acting independently. BLAST searches of the MGE7 locus against *M. galloprovincialis* whole genome shotgun sequence returned an alignment with contig mg10_S01094 (accession UYJE01010330.1) and 7 predicted *M. galloprovincialis* proteins VDI82194.1 ‐ VDI82200.1. Conserved domain searches revealed a similar structure to the transcriptional regulator Msx2‐interacting protein. The BLASTp search also returned significant alignments to Msx2‐interacting proteins in *Mytilus coruscus*, *Crassostrea virginica*, and *Haliotis rubra*. The existence of the MGE7 gradient highlights the role that environmental variation may play in retarding gene flow among wild *M. galloprovincialis* populations, and also how the success of collection of young mussels (spat) from one site and their transfer to another site (the farm) may be influenced by a single factor such as minSAL or maxSST on a localized scale.

## Graphical abstract

We investigated the genetic variation of the economically and ecologically important species the Mediterranean mussel (*Mytilus galloprovincialis*) from 18 sites along the eastern Adriatic Sea, using six microsatellite loci. Population genetic analysis of variation at five neutral microsatellite loci revealed little or no spatial variation. In contrast, seascape genetic analysis revealed a pronounced association between allelic and genotypic frequencies at a sixth locus, MGE7, and the environmental variable minimum salinity, highlighting the role that environmental variation may play in retarding gene flow among wild *M. galloprovincialis* populations.

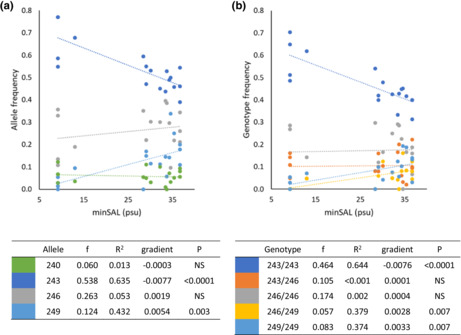

## 1 INTRODUCTION

Trying to understand the causative forces of patterns of population genetic variation at different spatial (and temporal) scales has long been a challenge for marine science (Schmidt et al., 2008; Selkoe et al., 2015). Classical population genetics is based on assumptions of marker neutrality because the investigator is usually trying to interpret gene flow (connectivity) in the absence of environmental variation – that is, without including the influence of selection because selective mortality may provide a picture of gene flow that is different from the actual (neutral) genetic connectivity (Holderegger et al., 2006). However, with the advent of highly polymorphic markers such as microsatellites, and more recently single nucleotide polymorphisms (SNPs), and with new analytical approaches, there is now potential to investigate how environmental variation may influence gene flow and population genetic structure (Gagnaire et al., 2015; Manel et al., 2003). The ability to generate new knowledge from markers that are themselves under selection (or are tightly linked to genes under selection) has implications for disciplines such as fisheries, aquaculture, and species conservation (Gaggiotti et al., 2009; Vaux et al., 2021; White et al., 2010), as well as providing new and broad insights into how organisms interact with their environments. For example, such new knowledge may be important in understanding species‐specific responses to environmental variation (e.g., increased sea surface temperature or changes in concentrations of aragonite/calcite and silica) in the face of climate change (e.g., Segovia et al., 2020; Wei et al., 2013a; Zeng et al., 2020).

For many benthic marine invertebrates, the main mechanism for exchange of individuals between populations is via a pelagic larval stage, the fate of which often depends on oceanographic features such as currents, fronts, straits, and shoreline configuration (Pascual et al., 2017; Riginos & Liggins, 2013). During the pelagic (dispersive) stage larvae may be transported within distinct packets of water, so environmental variables related to seawater may be relatively stable (Selkoe et al., 2006). With settlement in intertidal or shallow coastal regions where water mixing is often high, juveniles experience the sea moving about them bringing greater variation in environmental conditions (Riginos & Liggins, 2013). For sessile intertidal species, which cannot avoid the dynamic range of environmental stresses in the intertidal zone (Tomanek & Helmuth, 2002), the transition between pelagic and benthic stages may be a time of particular vulnerability (Pechenik, 1999). If extreme environmental conditions are a regular feature of the settlement location, selective mortality of juveniles can lead to genetic structuring in the adult populations. For example, peak spawning activity of Mediterranean mussels (*Mytilus galloprovincialis* Lamarck, 1819) in the Adriatic Sea occurs from late winter through to spring, a period that overlaps with times of pronounced freshwater influence (Da Ros et al., 1985; Krivokapić et al., 2011; Lipizer et al., 2014), a scenario that could lead to genetic structuring as a consequence of genotype‐dependent mortality in response to salinity fluctuation. This sort of environmentally driven selection has been very well documented in blue mussel (*Mytilus edulis* Linnaeus, 1758) populations in Long Island Sound (New York State, Atlantic coast of the USA), where regular annual selective mortality is driven by strong selection against juveniles with the *Lap* ^*94*^ allele in response to low salinity (Hilbish et al., 1982; Hilbish & Koehn, 1985).

A recent review of seascape genetics studies (Selkoe et al., 2016) noted that three factors – temperature, oceanography, and geography – were equally important in explaining patterns of spatial genetic variation across 100 studies. In addition, it was noted that many different factors are likely to affect connectivity at distinct spatio‐temporal scales, so while some studies may identify a single key (causative) environmental variable as explaining genetic variation, others will identify two or more variables, often acting synergistically (e.g., Silva & Gardner, 2016; White et al., 2010). It is increasingly clear that understanding patterns of spatial genetic variation, and the factors that contribute to the variation, is very much context‐ and species‐dependent. While important generalities may be drawn from reviews and meta‐analyses (e.g., Cárcamo, 2021; Selkoe et al., 2016), understanding the local situation is, of course, critical.

Our study focusses on *M. galloprovincialis*, a native species on the eastern shoreline of the Adriatic Sea. *M. galloprovincialis* is a model organism with a large body of research covering areas such as its role as an ecosystem engineer, physiological adaptations to the rocky intertidal zone, aquaculture and its potential as an invasive species (Arribas et al., 2014; Braby & Somero, 2006; Gardner et al., 2016; Kovačić et al., 2017; Wenne et al., 2022). Its success as an invader and ability to outcompete native congeners is in part due to its greater tolerance of a wide variety of environmental conditions in a time of global climate change (Branch & Steffani, 2004; Evans & Somero, 2010; Saarman et al., 2017; Tomanek, 2012). For example, on the Pacific coast of North America, it has supplanted the northern blue mussel (*Mytilus trossulus* Gould, 1850) in many locations from southern California (USA) to British Columbia (Canada) in waters where it is more warm‐tolerant than its native congener (Geller, 1999; Schneider & Helmuth, 2007; Tomanek, 2012).

**FIGURE 1:**
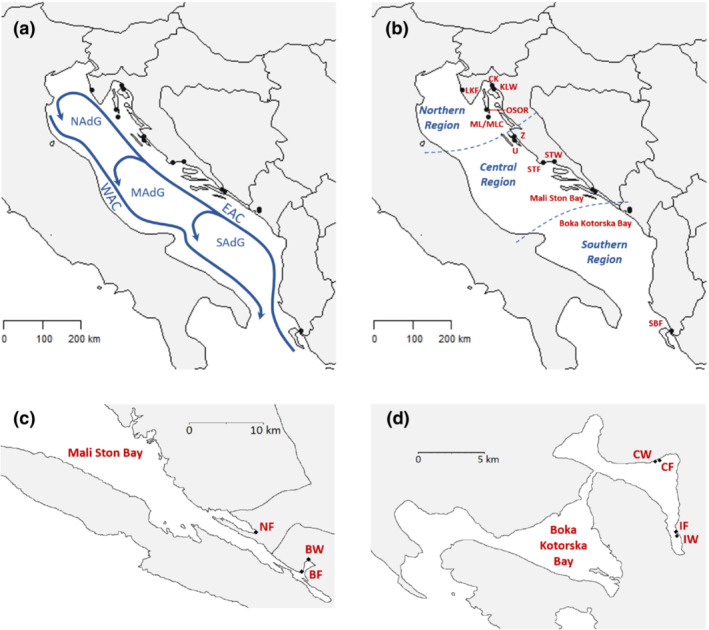
Sampling sites of mussels, *M. galloprovincialis*, showing (a) general currents: EAC eastern Adriatic current, WAC Western Adriatic current, NAdG northern Adriatic gyre, MAdG middle Adriatic gyre, SAdG southern Adriatic gyre (adapted from Lipizer et al., 2014), (b) the three regions used to test the hypothesis of genetic differentiation based on Adriatic Sea oceanography, (c) three sites in Mali Ston Bay, Bosnia & Herzegovina (NF) and Croatia (BF & BW), and (d) four sites in Boka Kotorska Bay (CW, CF, IF, IW), Montenegro. Site abbreviations as per Table 1.

The main circulation of the Adriatic Sea is cyclonic with the north‐flowing Eastern Adriatic Current (EAC) originating in the Ionian Sea and the Western Adriatic Current (WAC) flowing southward from the mouth of the Po River. Three cyclonic gyres, corresponding to northern, central, and southern basins, operate with varying strength through the year and significant variation between years may form barriers to larval dispersal and therefore lead to genetic discontinuities (Figure 1; Artegiani et al., 1997; Bray et al., 2017; Dubois et al., 2016; Zonn & Kostianoy, 2017). The Adriatic Sea is a dilution basin for the Mediterranean Sea, contributing ~30% of the freshwater inflow for the whole Mediterranean Sea (Estournel et al., 2021). The Adriatic Sea is nonetheless very saline with seasonal average salinity for the surface water of each of the three basins varying between 37.9–38.2, 37.7–38.3, and 35.5–37.4 psu for the southern, middle, and northern basins, respectively. Evaporation of water from the Adriatic Sea itself and the inflow of high‐salinity waters from the northern Ionian Sea (>38.6 psu) are the main processes that maintain this high salinity (Artegiani et al., 1997). The eastern Adriatic coastal region is characterized by a limestone karst landscape with accompanying erosion, which has led to an extensive and complex underground network of flow with many freshwater wells and springs, both on land and on the sea bottom (Bonacci et al., 2013). Mussels in these areas, as well as those in estuaries, can experience wide variations in salinity, which can change very quickly in times of heavy rain (UNEP/MAP‐RAC/SPA, 2015). Mussel tolerance to salinity variation may be as tied to the magnitude and speed of changes as to the absolute maximum and minimum values reached (Hamer et al., 2008).

The Mediterranean Sea is predicted to be particularly strongly influenced by global warming trends, with increasing occurrences of marine heat waves and temperature‐related mass mortality events (Di Camillo & Cerrano, 2015; Lejeusne et al., 2010; Michaelidis et al., 2014; Oliver et al., 2018). Conditions are already thought to be close to the upper limit of thermal tolerance for Mediterranean mussels in some regions with several instances of mass mortality associated with marine heatwaves in Spain, France, Greece, and in the Adriatic Sea (Galli et al., 2017; Lejeusne et al., 2010; Lupo et al., 2021; Michaelidis et al., 2014). Understanding existing levels of genetic diversity, patterns of gene flow, and the relationship between genetic variation, species ecology, physiology, and environmental factors is critical to the management of this ecologically and commercially important species. Understanding species biology in the native environment can also help in the management of the species in introduced habitats. Here, we describe the use of six polymorphic microsatellite markers to study the population and seascape genetics of 18 populations of *M. galloprovincialis*, along the eastern Adriatic coast.

## 2 MATERIALS AND METHODS

### 2.1 Sample collection

In total, 843 individuals of *M. galloprovincialis* were collected from 18 sites (hereafter populations) along the eastern coastline of the Adriatic Sea, a region spanning four countries (Croatia, Bosnia‐Herzegovina, Montenegro, and Albania) and a variety of coastal environments (Figure 1; Table 1). The 18 populations were divided between the three regions of the Adriatic Sea (defined by bathymetry and corresponding currents, see Figure 1) with six, seven, and five populations in the northern, central, and southern regions, respectively. Seven were farmed and 11 were wild populations. The number of individuals sampled per site varied between 31 and 50 (mean ± SD of 46.8 ± 5.6). A variety of different sizes (shell length) and therefore putative ages of mussels was collected per site, with shell length in the range of 8–85 mm. We collected all wild (native) mussels from the intertidal zone and the farmed mussels were collected from the shallow subtidal region (top 2 m of the water column at the farm). Samples of mantle tissue were preserved in 99% ethanol for DNA extraction.

**TABLE 1:** *Mytilus galloprovincialis* sampling site code and name, status (W, Wild; F, Farmed), sample size (*N*), collection date, and geospatial coordinates.

Site code	Site name	Wild (W) farmed (F)	Country	*N*	Collection date	Latitude	Longitude
LKF	Limski Channel	F	Croatia	31	09/2015	45.13334	13.66667
CK	Črišnjevo Krk bridge	W	Croatia	43	09/2015	45.24935	14.58243
KLW	Klimno, Soline Bay	W	Croatia	49	09/2015	45.15497	14.61889
OSOR	Cres‐Lošinj bridge	W	Croatia	35	09/2015	44.69283	14.39231
ML	Mali Lošinj Harbor	W	Croatia	50	09/2015	44.53228	14.46835
MLC	Mali Lošinj Čikat Bay	W	Croatia	50	09/2015	44.53181	14.45143
Z	Gaženica Harbor, Zadar	W	Croatia	50	09/2015	44.09786	15.25289
U	Ugljan‐Pašman bridge	W	Croatia	50	09/2015	44.01455	15.25086
STF	Poljica, near Split	F	Croatia	48	09/2015	43.51543	16.13917
STW	Vranjic, Split	W	Croatia	48	09/2015	43.53180	16.46667
NF	Neum, Mali Ston Bay	F	Bosnia & Herzegovina	50	09/2015	42.91312	17.62381
BF	Bistrina, Mali Ston Bay	F	Croatia	50	09/2015	42.86880	17.69835
BW	Bistrina, Mali Ston Bay	W	Croatia	50	09/2015	42.87976	17.70917
CF	Boka Kotorska Bay	F	Montenegro	50	09/2015	42.48539	18.74596
CW	Boka Kotorska Bay	W	Montenegro	50	09/2015	42.48568	18.74349
IF	Boka Kotorska Bay	F	Montenegro	50	09/2015	42.43705	18.76321
IW	Boka Kotorska Bay	W	Montenegro	44	09/2015	42.43596	18.76367
SBF	Butrint Lagoon	F	Albania	45	05/2017	39.75474	20.03251
Total				843			

### 2.2 Laboratory protocols

DNA was extracted using Geneaid genomic DNA Mini Kits following manufacturer's instructions and DNA concentrations were quantified using a NanoDrop™ ND‐1000 (Thermo Scientific). Individuals were genotyped using seven microsatellite loci described in three published papers as follows: Mgμ1 (Presa et al., 2002), MGE3, MGE4, MGE5, MGE7 (Yu & Li, 2007), MT203, MT282 (Gardeström et al., 2008). The choice of microsatellites was based on amplification success and the presence of polymorphism; details, including amplification conditions that followed Westfall (2011), are summarized in Table A1. Amplified fragments were scanned using an automated analyzer ABI 3730XL by two different service providers (Macrogen and Sangon Biotech).

### 2.3 Genetic data sets

Electropherograms were scored with Geneious 8.0.5 (http://www.geneious.com, Kearse et al., 2012) and Microchecker (Van Oosterhout et al., 2004) was used to check the microsatellite data for genotyping artifacts such as null alleles, large allele dropout, and stuttering. An outlier analysis to detect loci under selection was performed with Arlequin 3.5.2.2 (Excoffier & Lischer, 2010) using the Finite Island Model, 100 simulated demes and 20,000 coalescent simulations.

Different versions of the data set have been used for analyses (Table A2). This is because locus Mgμ1 had data missing for ~56% of all mussels and because locus MGE7 was potentially under selection (see outlier analysis section in Results). We used seven loci for all analyses, but repeated analyses with six loci (excluding Mgμ1). We present results for six loci only because results using seven loci and six loci (with and without Mgμ1) were similar. For the AMOVA, Structure and AWclust analyses, we also report results for five loci (i.e., excluding Mgμ1 and MGE7) so that results using neutral loci only may be compared directly to results that included MGE7. To calculate the *F*‐statistics used in the seascape analyses, we used a data set of 577 individuals containing complete data for six loci (i.e., excluding Mgμ1 because of missing data).

To assess whether sample sizes were sufficiently large to characterize the allelic variation of each population, the rarefaction procedure in the software package PopGenKit (Rioux Paquette, 2011) in R 3.5.1 (R Core Team, 2014) was used with 1000 jackknife replicates to estimate the total allelic diversity for each marker, each population and over all populations.

Analyses of Hardy–Weinberg equilibrium (HWE) and Linkage Disequilibrium (LD) were performed using Genepop 4.7.0 (Rousset, 2008). Departures from HWE were assessed by two methods, the probability test and the U‐test with the alternate hypothesis of heterozygote deficiency, using the default parameters. False discovery rate control for multiple statistical testing was applied to *p*‐values (Verhoeven et al., 2005).

The effective population size, *Ne*, was estimated using the linkage disequilibrium method of Hill (1981), Waples (2006), and Waples and Do (2010) as implemented in *Ne*Estimator 2.1 (Do et al., 2014). Rare alleles were excluded from the calculation as recommended by Waples and Do (2010).

### 2.4 Allelic and genotypic estimations and analyses

The frequency of alleles, number of private alleles (PA), observed and expected heterozygosities (H_O_ and H_E_), and the fixation index (*F*_IS_) were calculated using GenAlEx 6.503 (Peakall & Smouse, 2012). Allelic richness (A_R_) and private allelic richness (PA_R_) were calculated using HP‐RARE 1.1 (Kalinowski, 2005). Kruskal–Wallis tests were employed to test whether *F*_IS_ and A_R_ from farmed and wild populations were drawn from the same distribution. To test the hypothesis that alleles and genotypes are drawn from the same distribution in the 18 populations, exact *G*‐tests for population differentiation were performed in Genepop 4.7.0 for all populations using the default Markov chain parameters. The correlation between latitude and the frequency of the most common MGE7 allele was tested.

### 2.5 Differentiation among regions and populations

To test if there were genetic differences among populations in the three regions in the Adriatic Sea (Figure 1), a hierarchical analysis of molecular variance (AMOVA, Excoffier et al., 1992) was performed using GenAlEx 6.503 (Peakall & Smouse, 2012) for three regions and 18 populations with 999 permutations to determine statistical significance of the *F*_RT_ values. Within AMOVA, the *F*‐statistics were calculated for individual loci to find the contribution of each locus to the overall differentiation indices. The *p*‐values were corrected for family‐wise error using the False Discovery Rate formula. Pairwise population *F*_ST_ values were calculated to measure genetic differentiation between populations.

Two different cluster analysis programs, Structure (Pritchard et al., 2000) and AWclust (Gao & Starmer, 2008), were used to assess genetic structure for the 18 populations. Structure uses Bayesian analysis, which assumes conformation to HWE and LD assumptions, while AWclust is non‐parametric and therefore does not require conformity to HWE and LD assumptions. Structure analyses were performed with a burn‐in length of 50,000 steps, run length of 100,000 steps, 10 iterations for *K* = 1 to 18 using the correlated allele frequency model, and both the Admixture and No admixture models with sampling locations as priors. The Evanno method (Evanno et al., 2005) as implemented in the program Structure Harvester (Earl & vonHoldt, 2012) was used to detect the most appropriate number of clusters. AWclust analysis followed the allelic coding protocol (Gruber et al., 2013; Wei et al., 2013b) of 0 for no allele, 1 for heterozygous, and 2 for homozygous variants. To determine the most appropriate number of clusters, *K*, the gap statistic was calculated for *K* = 1–8 using 100 null simulations. For both Structure and AWclust, linear correlations were performed between latitude and the proportion of cluster membership for each population to see if cluster membership followed a latitudinal cline similar to MGE7 allele frequency.

### 2.6 Environmental data acquisition

Environmental data were collected from published articles and online databases (Table A3). Because there is no standardized environmental data collection between countries bordering the eastern Adriatic Sea, the data available varied widely among sites. Data from published articles were more plentiful for farmed and neighboring wild populations (LKF, NF, BF, BW, CF, CW, IF, IW, and SBF) and populations in large harbors (Z, STW, IF, IW) than for the other sites (CK, KLW, OSOR, ML, MLC, U, and STF) (see site abbreviations in Table 1). For these latter areas, which were all in Croatia, the online resources at SeaDataNet.org and the Croatian sea bathing water quality database (IOR, 2022), together with the models presented in Lipizer et al. (2014) and satellite data presented in Böhm et al. (2003), were used and also allowed for comparison among all sites. We did not use satellite data available from Copernicus because we could not retrieve data from enclosed bays and convoluted coastlines that accurately reflected fine‐scale conditions (Đurović et al., 2018). More detailed information about environmental data types, spatial and temporal coverage, and sources is provided in Hamilton (2019). Overall, there was good agreement for trends and inter‐site comparisons across all data sources, particularly for sea surface temperature and salinity data. The chlorophyll‐a data were not so complete, with detailed information available for the 10 southernmost populations but only rather sparse data for the eight northern populations, for which we relied on satellite‐derived data from Böhm et al. (2003) and the website SeaDataNet.org.

Nine environmental variables were used in analyses, the minimum and maximum values of sea surface temperature, salinity, and chlorophyll‐a concentrations (minSST, maxSST, minSAL, maxSAL, minCHL‐a and maxCHL‐a), together with delta values (deltaSST, deltaSAL and deltaCHL‐a – the difference between the maximum and minimum readings) that reflect the full range of variability. Three geospatial variables were also employed, latitude (Lat), longitude (Long), and the total coastal distance (TotalCD). TotalCD was calculated following Wei et al. (2013a) as the total of the pairwise coastal distances (CD in km) between any one population and all others (where CD is the shortest distance by sea between each population pair). Sites with high values of TotalCD are, on average, at a greater distance from all others than sites with low values of TotalCD. The environmental and geospatial variables used are detailed in Table A4.

### 2.7 Correlation testing of environmental/geospatial variables

To test for independence correlations between pairs of all twelve variables (nine environmental and three geospatial) were calculated and relationships visualized using principal component analysis using Statistica 7.1 (StatSoft). We removed variables so that |R| < 0.85 for all pairs of variables to avoid excess collinearity (e.g., Anderson et al., 2008).

### 2.8 Seascape genetics ‐ GLZ analysis

A generalized linear model (GLZ in Statistica) was used to describe the relationship between a genetic response variable and site‐specific environmental variation. GLZ analysis was carried out for five separate metrics: (1) Lin*F*_ST5_ (i.e., MGE3, MGE4, MGE5, MT203, MT282) which is based on the complete data set of five neutral loci, (2) Lin*F*_ST6_ (i.e., MGE3, MGE4, MGE5, MT203, MT282 plus MGE7) which includes the locus that is putatively under selection, (3) Lin*F*_ST_MGE7 which was based only on the MGE7 locus only, (4) f(MGE7^243^) ‐ the site‐specific frequency of allele MGE7^243^, and (5) f(MGE7^243/243^) ‐ the site‐specific frequency of the homozygous genotype. Estimates of linearized *F*_ST_ (Lin*F*_ST_) were calculated using GenAlEx 6.503 (Peakall & Smouse, 2012) following Wei et al. (2013a). These metrics focus progressively on the MGE7 locus and the 243 bp allele, starting with a measure containing no information from the MGE7 locus and finishing with measures focused on the frequency of the 243 bp allele and homozygote. GLZ analyses were carried out using six predictor (independent) variables: minSAL, minSST, maxSAL, minCHL‐a, Lat, and TotalCD.

GLZ with a normal distribution and a Log‐link function was employed to identify the best‐fit model based on the Akaike information criterion (AIC). For each of the five genetic indices, the top‐ranked model (lowest AIC value) and a set of best‐fitting models were used to evaluate which of the individual or combined predictor variables were most important in explaining variation in the genetic index. The number of models selected for the set of best‐fitting models was chosen by inspecting the plot of AIC values against the number of models and finding a point of inflection in the plot. This inflection is the point at which successive models add only a small and relatively constant amount to the AIC and are therefore not additionally informative (this is equivalent to the inspection of a scree plot for a PCA). The importance of each independent variable in explaining genetic structure was evaluated by calculating the percentage of best‐fitting models containing that independent variable (Figure A1; Table A5).

### 2.9 Seascape genetics ‐ distance‐based linear modeling

To test the effect of the six independent environmental/geospatial variables (minSAL, minSST, maxSAL, minCHL‐a, Lat, TotalCD) on site‐specific genetic variation, a distance‐based linear model was employed (e.g., Silva & Gardner, 2016). The DISTLM routine (PRIMER v6 with PERMANOVA add‐on ‐ Anderson et al., 2008) was used to test for associations between genetic and environmental variation (9999 permutations). The environmental and geospatial data were normalized before use. Genetic data were not transformed (i.e., raw population‐specific allele frequency data were employed) and a Euclidean distance similarity matrix was generated. Because this is a permutational test there are no assumptions about data normality (Anderson et al., 2008). The contribution of individual variables to explaining variation in the genetic data set was tested using marginal tests. Then, a model building approach was employed that tests all possible models and that identifies the best‐fit model containing successively more variables based on the Akaike Information Criterion (AIC), while also providing information about greatest improvement of the correlation coefficient (*R* ^2^). DISTLM analysis was carried out on the 6‐locus data set (i.e., excluding Mgμ1).

### 2.10 Correlation of MGE7 alleles and genotypes with environmental variables

We tested MGE7 alleles and genotypes for correlation with environmental variables that were important in explaining population‐specific genetic variation in the GLZ and DISTLM analyses. We tested correlations for all alleles and genotypes with frequencies greater than 0.05 (four alleles ‐ MGE7^240^, MGE7^243^, MGE7^246^, MGE7^249^ and five genotypes ‐ MGE7^243/243^, MGE7^243/246^, MGE7^246/246^, MGE7^246/249^, MGE7^249/249^) and also for the heterozygote genotype MGE7^243/X^ where X is any other allele.

## 3 RESULTS

### 3.1 Genetic data and use of different data sets

Six of seven microsatellite loci were amplified satisfactorily with 85.1%–98.7% of individuals scored. Locus Mgμ1 showed stuttering and multiple peaks for a proportion of samples, with 44.1% of individuals able to be reliably scored overall and the percentage scored per population ranging between 28.0% and 64.7%. Because results of analyses with and without this unreliable locus were similar, we have only reported results not including this locus. There was no relationship between percentage scored per population and population latitude (*R* ^2^ = .0004, *p* = .94). The percentage of each locus scored per population was broadly consistent between the two service providers.

The outlier analysis did not detect any significant deviations in the *F*_ST_ vs. heterozygosity among the loci. The high *F*_ST_ value of the MGE7 locus, together with results from correlation testing, AMOVA, Structure, and AWClust, which clearly show MGE7 behaving differently from the other loci, did however suggest that MGE7 might in fact be an outlier that was not identified statistically because of the low number of loci used. We interpret this conservatively and suggest that this locus may be under selection or is located close to a gene under selection. As a consequence, we present analyses for the neutral (five loci, excluding MGE7) and the six locus data set (including MGE7).

The application of Microchecker identified an excess of homozygote genotypes across all 18 populations for four of the six loci (MT282, MGE4, MGE7, and MT203). For the remaining two loci, MGE3 and MGE5, only one and three populations, respectively, had excess homozygotes. All 18 populations showed significant departures from HWE expectations with four individual loci being out of HWE at every population. Loci MGE3 and MGE5 met expectations at 15 and 11 populations, respectively. There was no evidence of linkage disequilibrium for any pair of loci, either at population level or across all populations.

Rarefaction curves showed that ~80% of allelic variation was captured in each population for six loci estimates of the effective population size, *Ne*, were large (Table A6). For the majority of populations, the upper limit of the 95% confidence interval was “infinite,” where an “infinite” population size means that there is no evidence of genetic variation caused by genetic drift due to a finite number of parents. Only one population, IW, had a finite upper limit for the 95% confidence interval for both data sets.

### 3.2 Allelic and genotypic estimations and analyses

Average allelic richness, A_R_, ranged from 6.16 in NF to 7.90 in CK, and private allelic richness, PA_R_, ranged from 0.00 in SBF to 0.40 in STF. In total, there were 15 private alleles. Observed heterozygosity, *H*_O_, expected heterozygosity, *H*_E_, and the inbreeding coefficient, *F*_IS_, was similar for the six and seven locus data sets. *H*_O_ was lower than *H*_E_ at every population, with *H*_O_ = 0.389 and *H*_E_ = 0.629 across all populations (using six loci). This is reflected in the high inbreeding coefficient, *F*_IS_, for which values ranged from 0.267 to 0.453 across all populations and was 0.365 overall (Table A7). The individual *F*_IS_ for each locus averaged over all populations varied considerably. Loci with low levels of homozygote excess (MGE3 and MGE5) had lower *F*_*IS*_, 0.052 and 0.115, respectively, compared to the loci that did have excess homozygotes at every population (MT282, 0.583; MGE4, 0.483; MGE7, 0.601; and MT203, 0.365).

The probability that alleles from each population were drawn from the same distribution for six loci, calculated by exact *G*‐tests, was <1 × 10^−20^ across all loci, less than 1 × 10^−5^ for individual loci MT282, MGE4, MGE7, and MT203, 0.021 for MGE5 and 0.187 for MGE3. The probability that genotypes from each population were drawn from the same distribution for six loci was 4.4 × 10^−7^ across all loci, less than 1 × 10^−5^ for MGE7, 0.018 for MT282 and not significant for the remaining loci.

**FIGURE 2:**
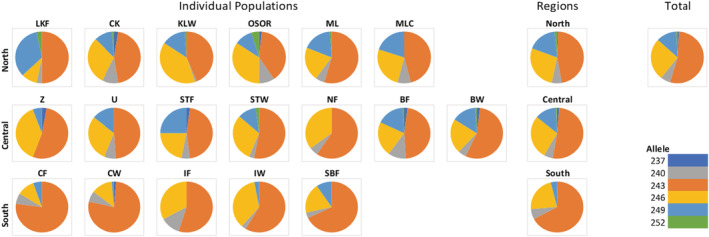
Allele frequencies for MGE7 for each population, each region and across all data.

**FIGURE 3:**
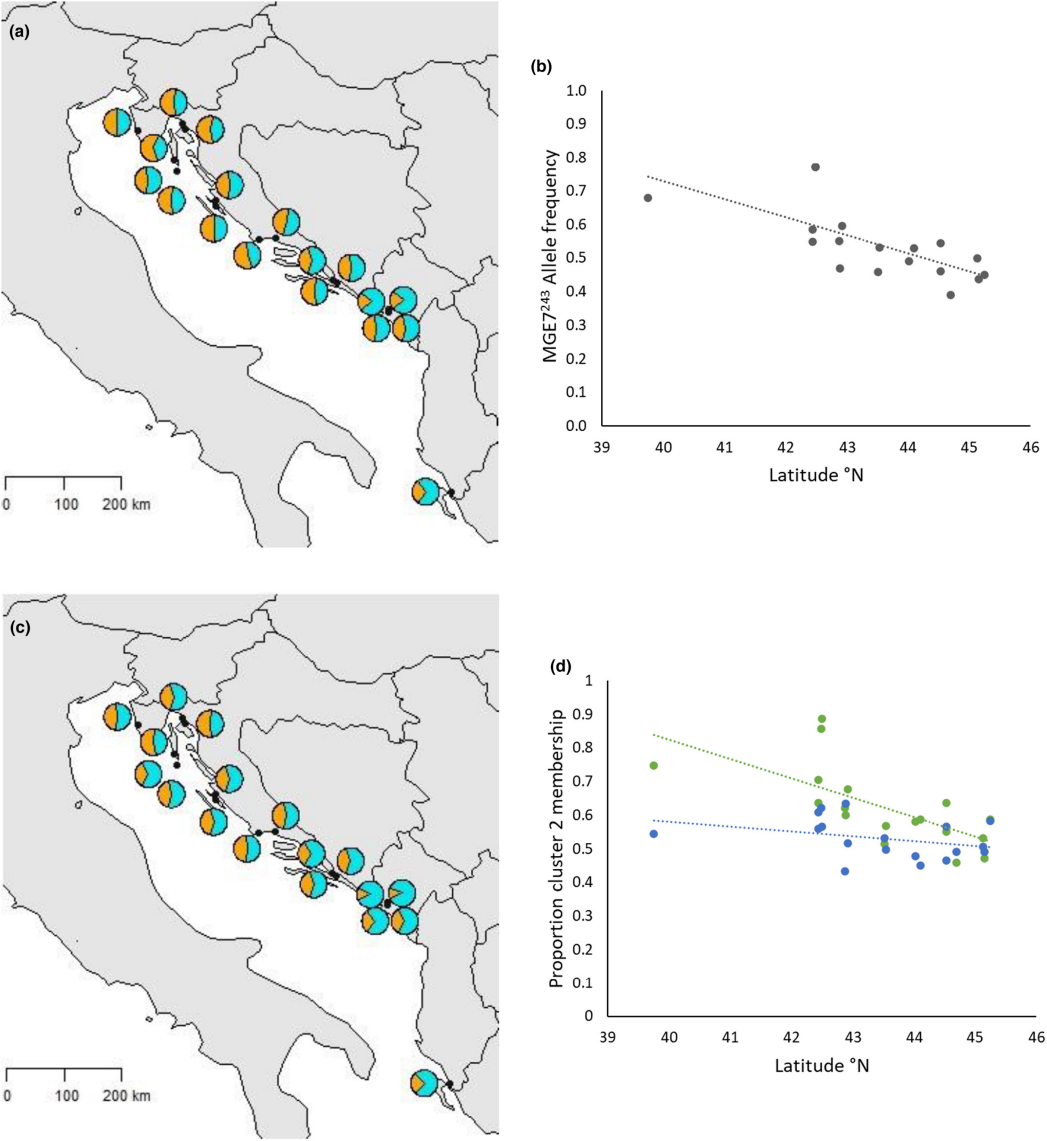
MGE7^243^ allele frequency and structure results: (a) map of frequency of MGE7^243^ allele (cyan) and all other alleles (orange) at each population, (b) correlation of frequency of MGE7^243^ allele with latitude, (c) map of proportion of cluster membership for six loci from the structure analysis (cluster 1 orange and cluster 2 cyan), (d) correlation of proportion of cluster membership by population for six loci (green) and five neutral loci (blue).

There were no differences in A_R_ or *F*_IS_ between farmed and wild populations (Kruskal‐Wallis *H* statistic = 0.740, *p* = .39 and *H* statistic = 2.628, *p* = .10 for A_R_ and *F*_IS_, respectively). Regional allele frequencies are illustrated in Figure A2. The frequency of the most common allele of the MGE7 locus, 243 bp in length, MGE7^243^, showed an increasing southward trend, with average frequency 46.5% in the North (range 39.1%–54.4%), 51.4% in the Central region (range 45.8%–59.5%), and 66.9% in the South (range 54.9%–77.0%) (Figure 2). The frequency of MGE7^243^ at each population was negatively correlated with latitude (*R* ^2^ = .476, *p* = .0015) (Figure 3).

### 3.3 Differentiation among regions and populations

AMOVA (Table 2) revealed small but significant genetic differentiation among the regions for all six loci (*F*_RT_ = 0.0031, *p* < .001) but not for the five neutral loci (*F*_RT_ = 0.0007, *p* = .085). The *F*_ST_ index, which in the hierarchical AMOVA is an index of variation explained between regions and populations to total variation, was significant for all six loci (*F*_ST_ = 0.008, *p* < .001) and also for the five neutral loci (*F*_ST_ = 0.002, *p* = .024).

**TABLE 2:** Hierarchical AMOVA and differentiation indices using six loci and five neutral loci, for three regions and 18 populations

Source of variation	Six loci					Five neutral loci				Index
	Df	SS	Est var	% var	Index	Df	SS	Est var	% var	
Among regions	2	14.7	0.006	0.31%	*F*_RT_ = 0.0031 *p* < .001	2	6.3	0.001	0.07%	*F*_RT_ = 0.0007 *p* = .085
Among populations	15	58.4	0.010	0.52%	*F*_SR_ = 0.0052 *p* < .001	15	37.8	0.002	0.12%	*F*_SR_ = 0.001 *p* = .108
Among individuals	817	2387.9	0.909	44.7%	*F*_ST_ = 0.0082 *p* < .001	817	1850.4	0.665	39.69%	*F*_ST_ = 0.002 *p* = .024
Within individuals	835	923.0	1.105	54.4%	*F*_IS_ = 0.451 *p* < .001	835	815.5	1.007	60.12%	*F*_IS_ = 0.398 *p* < .001
			2.031	100.0%	*F*_IT_ = 0.456 *p* < .001			1.675	100.00%	*F*_IT_ = 0.398 *p* < .001

Abbreviations: % var, percentage variation; Df, degrees of freedom; Est var, estimated variation; SS, sum of squares.

For the six locus data set, all three pairwise comparisons of *F*_RT_ values among regions were statistically significant (*p* ≤ .012) whereas for the five locus data set only the North‐Central comparison was significant (Table 3).

**TABLE 3:** Pairwise *F*_RT_ for comparisons between the three regions are below the diagonal with *p*‐values following 9999 permutations above the diagonal

Six loci				Five loci			
Region	North	Central	South	Region	North	Central	South
North		0.012	≤0.0001	North		0.013	0.146
Central	0.001		≤0.0001	Central	0.002		0.376
South	0.008	0.004		South	0.001	0.000	

*Note*: Results for all six loci are on the left and for the five neutral loci on the right.

Pairwise population *F*_ST_ values for the six locus and five neutral locus data sets were variable with 64 of 153 comparisons significant after FDR correction for the six locus data set and only one for the neutral data set (Table A8). Two populations exhibited unusually high *F*_ST_ values for the neutral data set, ML and Z, both with mean an *F*_ST_ value of 0.008 compared to mean values of 0.001 to 0.004 for all other population pairwise comparisons.

The Structure analysis runs using the Admixture model for the six locus data set had a maximum likelihood statistic, Δ*K*, at *K* = 5, with a local maximum at *K* = 2. When the No Admixture model was used, the maximum likelihood was attained at *K* = 2 (Figure A3). The Structure runs for *K* = 5 did not show any obvious genetic structure but for *K* = 2, using both models, the bar plot suggested a north–south cline in the proportion of ancestry from each cluster, something that was confirmed by linear correlation tests of proportion of cluster membership as a function of latitude (*R* ^2^ = .459, *p* = .002, Figures 3 and A3). Structure runs on the neutral data set did not show this north–south cline (*R* ^2^ = .111, *p* = .178, Figure 3) and there was no evidence of any genetic differentiation.

AWclust analyses (Figure A4) of the six locus data set revealed overlapping confidence intervals and local maxima at *K* = 2, 4, and 7, indicating weakly defined structure. For *K* = 2, the correlation between cluster 2 membership and latitude was significant (*R* ^2^ = .308, *p* = .017) but weaker than the corresponding correlation from the Structure program. For the five neutral loci, only one cluster was evident and when *K* = 2, the correlation between cluster 2 membership and latitude was not significant (*R* ^2^ = .073, *p* = .279).

### 3.4 Correlation testing of environmental/geospatial variables

Based on PCA and correlation analysis with a critical cut‐off value of |R| > 0.85, we removed six of 12 environmental and geospatial variables from the data set (Figure A5, Table A9). Subsequent analyses involved six independent environmental and geospatial variables (minSAL, minSST, maxSAL, minCHL‐a, Lat, and TotalCD).

### 3.5 Seascape genetics ‐ GLZ analysis

GLZ analyses were performed on five genetic response indices ‐ Lin*F*_ST5_, Lin*F*_ST6_, Lin*F*_ST_MGE7, f(MGE7^243^), and f(MGE7^243/243^) (Table 4). The top‐ranked models became more significant statistically as the genetic response index honed in on the MGE7^243^ allele: *p* = .177 for neutral loci (Lin*F*_ST5_), *p* = .071 for all loci including MGE7 (Lin*F*_ST6_), *p* = .023 for MGE7 only (Lin*F*_ST_MGE7), *p* < .0001 for both allele frequency (f(MGE7^243^)) and genotype frequency (f(MGE7^243/243^)). The variable minSAL was the most important of all predictor variables in explaining genetic variation: it was included in the top‐ranked model for all genetic indices except Lin*F*_ST5_ and had a significant all effects test for all genetic indices except Lin*F*_ST_MGE7. It was included in an increasing percentage of the best‐fitting models as the genetic index focused on the MGE7^243^ allele (it was included in 12.5% of best‐fitting models for Lin*F*_ST5_, 70% for Lin*F*_ST6_, 73.3% for Lin*F*_ST_MGE7, 100% for both f(MGE7^243^) and f(MGE7^243/243^)). Although minSST and minCHL‐a were included in the top‐ranked model (together with minSAL) for f(MGE7^243/243^), the three lowest ranked models of all possible models included only minSST and/or minCHL‐a.

**TABLE 4:** Results from GLZ analysis showing the top‐ranked models for variation in five genetic indices, Lin*F*_ST5_, Lin*F*_ST6_, Lin*F*_ST_MGE7, f(MGE7^243^), and f(MGE7^243/243^).

Genetic index	Top‐ranked model		Best‐fitting models						Intercept *p*
	Model	*p*	minSAL	minSST	maxSAL	minCHL‐a	Lat	TotalCD	
Lin*F*_ST5_ neutral loci	minSST	.177	**12.5**	**50**	25	0	25	25	.014
Lin*F*_ST6_ six loci	minSAL Lat	.071	**70**	30	30	10	**80**	20	.010
Lin*F*_ST_ MGE7	minSAL Lat	.023	73.3	53.3	53.3	13.3	**73.3**	26.7	.143
f(MGE7^243^)	minSAL	<.0001	**100**	28.6	28.6	14.331.6	14.3	14.3	.400
f(MGE7^243/243^)	minSAL minSST minCHL‐a	<.0001	**100**	**80**	40	46.7	33.3	26.7	.108

*Note*: The top‐ranked model for each test is shown with *p*‐value, together with the percentage of each variable included in the set of best‐fitting models: Significant values according to the all‐effects test shown in bold. The *p*‐values for the all‐effects test on the intercept (null hypothesis: The intercept is at zero) are also shown.

### 3.6 Seascape genetics ‐ distance‐based linear modeling

Marginal analyses revealed that two of the six variables explained significant variation in the 6‐locus data set (minSAL (*p* = .0016), Lat (*p* = .0434)) (Table 5). Model building based on AIC values revealed that the single best‐fit model contained only one variable – minSAL (Table 5). This model explained 13.8% of the variation in the genetic data set. As expected, addition of new terms to the model improved the fit as judged by the *R* ^2^ value, which increased from 0.138 (one variable) to 0.432 (six variables). The variable minSAL was included in all six of the six best‐fit models, indicating that it is the single variable with greatest power to explain variation in the genetic data set.

**TABLE 5:** Results of the distance‐based linear modeling (DISTLM) analyses, including marginal tests (per individual variable) and best‐fit models for all possible number combinations of variables.

Marginal tests			
Variable	Pseudo‐*F*	*p*	Proportion
minSAL	2.560	.0016	0.138
Lat	1.776	.0434	0.100
maxSAL	1.490	.1035	0.085
minCHL‐a	1.067	.3372	0.062
minSST	0.933	.5293	0.055
TotalCD	0.762	.6624	0.045

Best models ‐ best result for each number of variables			
No. of variables	AIC	*R* ^2^	Variables selected to be in the model in model
1	−38.543	.138	minSAL
2	−37.630	.188	minSAL + minCHL‐a
3	−37.434	.266	minSAL + minSST + maxSAL
4	−36.893	.323	minSAL + minSST + maxSAL + Lat
5	−36.768	.390	minSAL + minSST + maxSAL + Lat + TotalCD
6	−36.064	.432	minSAL + minSST + maxSAL + Lat + TotalCD + minCHL‐a

### 3.7 Correlation of MGE7 alleles and genotypes with environmental variables

**FIGURE 4:**
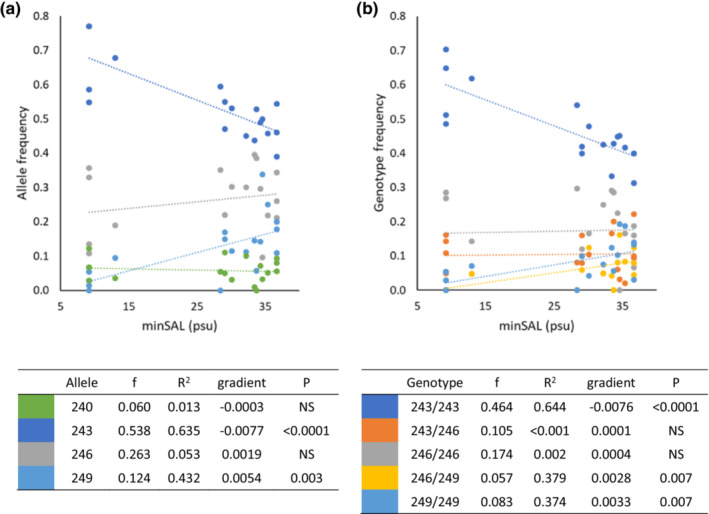
(a) Correlation of MGE7 allelic frequencies with minSAL, for alleles with overall frequency >0.05, showing frequency (f) of each allele over all populations, the correlation coefficient (*R* ^2^), gradient, and *p*‐value. (b) Correlation of MGE7 genotypic frequencies with minSAL, for genotypes with overall frequency >0.05, showing frequency (f) of each genotype over all populations, the correlation coefficient (*R* ^2^), gradient, and *p*‐value.

The most common allele over all populations, MGE7^243^, was negatively correlated with minSAL (*f* = 0.538, *R* ^2^ = .635, *p* < .0001). This was partially counterbalanced by the positive correlation of MGE7^249^ (*f* = 0.124, *R* ^2^ = .432, *p* = .003). The second most common allele, MGE7^246^, was not correlated with minSAL (*f* = 0.263, *R* ^2^ < .001, *p* = NS) (Figure 4).

The MGE7^243/243^ homozygous genotype was also negatively correlated with minSAL (*f* = 0.464, *R* ^2^ = .644, *p* < .0001), while there was no correlation between the heterozygous genotype, MGE7^243/246^ and minSAL. Both MGE7^246/249^ (*f* = 0.057, *R* ^2^ = .379, *p* = .007) and MGE7^249/249^ (*f* = 0.0.083, *R* ^2^ = .374, *p* = .007) were positively correlated with minSAL (Figure 4). Although the observed frequency of the MGE7^243/249^ genotype was 0.009 (*n* = 7 mussels observed), the expected frequency was 0.13 (*n* = 100.6 mussels expected). The ratio of observed to expected for this genotype (0.07) was very low, even in a background of heterozygote deficiencies. The ratios of observed to expected for the three other heterozygous genotypes with expected frequency >0.05, MGE7^243/246^, MGE7^240/243^, and MGE7^246/249^, were 0.37, 0.50, and 0.87, respectively.

The variables minSST and minCHL‐a were included with minSAL in the top‐ranked model for the GLZ analysis based on the genetic response index f(MGE7^243/243^) but neither was significantly correlated with any MGE7 allele or genotype.

## 4 DISCUSSION

In this study, focusing on 18 populations of *M. galloprovincialis* along the eastern coast of the Adriatic Sea, neutral multi‐locus microsatellite analysis revealed low levels of genetic differentiation, very large population sizes and heterozygote deficiencies (consistent with a large body of work, e.g., Daniels & Litvaitis, 2017, Hedgecock et al., 2004, Wei et al., 2013b, Zouros & Foltz, 1984). These results add fine‐scale detail to the literature and are consistent with other regional or basin‐wide studies that showed either well‐connected populations in the Adriatic Sea close to panmixia, or weak east–west and/or north–south differentiation (Giantsis et al., 2014; Paterno et al., 2019; Štambuk et al., 2013; Wenne et al., 2022). We also report a pronounced gradient in allelic and genotypic frequencies at one locus that is very strongly associated with environmental variation along the eastern Adriatic Sea coastline.

The potential role of selection in generating a gradient of genetic variation is supported by the seascape analyses that revealed a strong association between the frequency of allele MGE7^243^ and minSAL. In the GLZ modeling, the associations between genetic variation and minSAL increased in strength as the information included in each genetic index honed in on the occurrence of the MGE7^243^ allele. While models using the genetic index based on neutral loci (Lin*F*_ST5_) were not statistically significant and minSAL was not included in the top‐ranked model, the measures that included the MGE7 locus (Lin*F*_ST6_ and Lin*F*_ST_MGE7) were strongly associated with the geospatial variable Lat and, to a lesser extent, minSAL. The genetic indices based solely on the frequency of the allele MGE7^243^ and on the homozygous genotype MGE7^243/243^ were strongly associated minSAL, and the models were statistically highly significant (*p* < .0001). The geospatial variable Lat was not statistically significant in these multivariate models, this result being consistent with the stronger single variable correlation of MGE7^243^ allele frequency with minSAL (*R* ^2^ = .635, *p* < .001) than with Lat (*R* ^2^ = .476, *p* = .015). The association between the MGE7^243^ allele and minSAL extended to the homozygous MGE7^243/243^ genotype, but not to the heterozygous MGE7^243/X^ genotype. Analysis of regional subsets of the data (e.g., northern, central, southern Adriatic) and analysis with or without individual sites (e.g., farmed and wild samples from Boka Kotorska Bay) revealed that the overall relationship is robust and not dependent on the inclusion or exclusion of specific sites (results not shown). Variations in allele frequencies that coincide with changes in an environmental variable or geographic cline may arise from chance effects and historic events (Schmidt et al., 2008), but the distinctive distribution pattern of the homozygous and heterozygous genotypes is much less likely to arise by chance alone and suggests a selective pressure. The MGE7 locus may be a neutral hitchhiker locus situated close to a gene under selection or it may itself be part of the coding region of a gene under selection (Gagnaire et al., 2015). This would not be surprising, since the locus was derived from EST sequences from GenBank (Yu & Li, 2007); it was however unexpected since this locus has previously been used successfully as a neutral marker (Westfall, 2011), including in one study focused on the Croatian coast (Štambuk et al., 2013). The trend of decreasing frequency of the MGE7^243^ allele with increasing minimum salinity is not balanced equally by the remaining alleles. The second most common genotype, MGE7^246/246^, is not correlated with minSAL, while less common genotypes involving the MGE7^249^ allele such as MGE7^246/249^ and MGE7^249/249^ are positively correlated (*R* ^2^ = .379 and 0.374, respectively, *p* = .007 for both). The MGE7^243^ allele appears to confer an advantage in low‐salinity environments only when alleles MGE7^246^ and MGE7^249^ are not also present as the heterozygote combination, while MGE7^249^ may be advantageous in high‐salinity environments either as a homozygote or heterozygote. This is supported by the low frequency of the MGE7^243/249^ genotype which was lower than expected even in a background of heterozygote deficiency.

For the locus MGE7, we see a complex picture of relationships between the different genotypes and the environmental variable minSAL and interactions between alleles which suggest that the locus, or a nearby gene, may be under selection with two– three different alleles. A BLASTn search of the MGE7 locus against *M. galloprovincialis* whole genome shotgun contigs returned an alignment with contig mg10_S01094 (accession UYJE01010330.1) and a BLASTp search found 7 predicted proteins VDI82194.1 ‐ VDI82200.1. The 7 coding sequences span nucleotides 66,989–122,497 of UYJE01010330.1 with VDI82194.1 and VDI82195.1 covering the entire range. A conserved domain search retrieved 4 RNA recognition motif (RRM) superfamily domains (cl17169) close to the N‐terminal and a SPOC SHARP domain (cd21543) close to the C‐terminal. This is similar to the structure of the SHARP protein (also known as Msx2‐interacting protein) which has 3 N‐terminal RRM domains and a C‐terminal SPOC SHARP domain (Arieti et al., 2014). The BLASTp search also returned significant alignments to Msx2‐interacting proteins in other mollusks, including the mussel *Mytilus coruscus*, the oyster *Crassostrea virginica* and the abalone *Haliotis rubra*. Msx2 is a transcriptional regulator with a well‐documented role in the regulation of cadherins, molecules involved in cell–cell adhesion (e.g. Liang et al., 2016). Cadherins, in turn, are involved in the response to hyposalinity in the eastern oyster, *Crassostrea virginica* (Jones et al., 2019). While the link between MGE7 and cadherin regulation is somewhat tenuous, it does suggest a possible mechanism for our hypothesized link between MGE7 variants and salinity. However, the correlation of the variables minSAL and maxSST (R = −0.911) and consequent removal of maxSST from the seascape analyses means that firm conclusions about which of the variables is most important to the mechanism of the putative selection cannot be made. The correlation can be characterized by a progression from island sites close to the open sea (OSOR, ML, MLC) with high minimum salinity and mild summer temperatures, through to sites in enclosed bays and lagoons (CF, CW, IF, IW in Boka Kotorska Bay and SBF in Butrint Lagoon) with high freshwater inputs and/or low water exchange with the sea (low minimum salinity) and high summer temperatures (Bellafiore et al., 2011; Moisiu et al., 2016). Intermediate sites include those with low (LKF, CK, KLW, Z, U, and STF) and moderate (STW, NF, BF, and BW) levels of freshwater influence and moderate summer temperatures.

Both maxSST and minSAL are relevant to many biological processes that may affect individual survival, and have long been known to play roles in genetic selection related to environmental clines or habitat mosaics (e.g., Gardner & Palmer, 1998; Halpin et al., 2002; Hilbish & Koehn, 1985; Logan et al., 2012; Negri et al., 2013; Wenne et al., 2020). While some differences in tolerance to thermal and hypoosmotic stresses are linked to differences at the individual gene level (such as in the *Lap* gene of *M. edulis* in Long Island Sound, New York State, Hilbish et al., 1982), other differences are at the regulatory level of transcription or post‐translational modification. In an analysis of the transcriptional response of *Mytilus* congeners to acute heat and low‐salinity stress, of 45 genes that responded to both stressors, the response was in opposite directions for 44 of the genes, with the one gene with a response in the same direction encoding a thioredoxin reductase enzyme involved in oxidative stress (Lockwood et al., 2015). Although relatively few changes in transcriptional regulation between *M. galloprovincialis* and *M. trossulus* resulted in substantial differences in thermal tolerances between the congeners (Lockwood et al., 2015), the severe impact of heat stress on highly evolved and conserved systems such as energy metabolism and detoxification argue that there is little room for further evolutionary adaptation to thermal stress (Michaelidis et al., 2014; Tomanek, 2012).

Environmental stresses, including thermal and hyposaline stress, lead to increased levels of oxidative stress, thus if selection is the driving force behind the variation in MGE7 allele frequencies, the mechanism may be related to pathways that deal with oxidative stress rather than mechanisms specifically related to low salinity or high temperature (Hamer et al., 2008; Michaelidis et al., 2014). The effects of environmental stresses operate synergistically to increase overall levels of oxidative stress, thereby reducing tolerance to individual stresses. For example, heavy metal pollution increases sensitivity to thermal stress (Michaelidis et al., 2014). Environmental stresses increase energy demands generally in order to maintain cellular homeostasis, repair damaged proteins, and detoxify reactive oxygen species (ROS). There is a trade‐off between energy metabolism which generates ROS and expending energy to detoxify ROS, which can reach a tipping point particularly during times of thermal stress when food availability may be at its lowest (Michaelidis et al., 2014). Environmental stresses also increase susceptibility to disease which may trigger mass mortalities (Di Camillo & Cerrano, 2015; Lejeusne et al., 2010). Any adaptive mechanism that enhances the ability of mussels to respond to oxidative stress is expected to be favored in environments which experience extremes in both sea surface temperature and salinity (e.g., Boka Kotorska Bay and Butrint Lagoon, the locations of sampling sites CF, CW, IF, IW, and SBF) (Hamer et al., 2008, Michaelidis et al., 2014).

A limitation of this study is the small number of microsatellite loci available for use. Surprisingly for such an ecologically and economically important species group, there has been only a limited number of microsatellite loci developed for use with the *Mytilus* species complex (Araneda et al., 2016; Diz & Presa, 2008, 2009; Giantsis et al., 2014; Larraín et al., 2015), and a limited number of studies investigating population genetic structure of *M. galloprovincialis* using microsatellites. Interestingly, markers have often not performed well when used on mussels from different geographic regions, with, for example, only a subset of markers developed for western Mediterranean mussels (Diz & Presa, 2008), performing satisfactorily for eastern Mediterranean mussels (Giantsis et al., 2014; Štambuk et al., 2013). We suspect that this may be because of the regional differences that appear to exist in the pan‐genome of *M. galloprovincialis* (Gerdol et al., 2020), which also may explain the very high proportion of null alleles (i.e., via hemizygosity) that is so often reported for this species and why so many loci are out of HWE. More recently population genetic analyses have been performed using SNPs (Gardner et al., 2016; Zbawicka et al., 2014) including the Mediterranean and Black Seas (Paterno et al., 2019; Wenne et al., 2022). Our data are consistent with the SNP results but add to our understanding by being much more focussed geographically and using, where possible, local environmental data. Although SNPs are now the marker of choice (Fischer et al., 2017), microsatellites offer complementary information to SNPs, having a higher mutation rate and more recent temporal scale of inference (Fotsing et al., 2019; Waits & Storfer, 2015; Wu et al., 2021).

The findings of this study have important implications for aquaculture and conservation management in relation to climate and other anthropogenic change. Aquacultural practice along the eastern Adriatic coast is largely traditional with spat often transferred from natural spawning grounds to aquaculture sites with favorable conditions for growth (Giantsis et al., 2014; Kovačić et al., 2017). The traditional methods of mussel farming employed are extremely labor intensive so the matching of environmental conditions between the natal site of the spat and the aquaculture site is recommended to reduce selective mortality related to environmental stresses (Mandić et al., 2017; Ramón et al., 2007). This is illustrated by the MGE7 locus, with the frequency of genotypes differing between environments. Aquaculture sites are often in bays and lagoons (for example, sampling sites CF and IF in Boka Kotorska Bay and SBF in Butrint Lagoon) with high maxSST and low minSAL. Our results suggest that juveniles collected from areas of low environmental variability will have lower proportions of the favorable MGE7^243^ allele than juveniles collected from areas of high environmental variability and are more likely to undergo selective mortality.

Climate change has altered the geographic range of many species, moving ranges poleward and has been implicated in the success of *M. galloprovincialis* as an invasive species outside of its native range (reviewed by Gardner et al., 2021). Within its native range of the Mediterranean Sea, reports of mass mortalities linked to extended periods of elevated temperatures are increasing (Galli et al., 2017; Lejeusne et al., 2010; Michaelidis et al., 2014; Verdura et al., 2019). For example, widespread disease triggered by long‐lasting high water temperatures and eutrophic waters in the northern Adriatic Sea led to mortality across multiple taxa, including *M. galloprovincialis*, which, in some areas, decreased in abundance from ~20% coverage to ~2% in 2013, with recovery to 9.5% by 2014 (Di Camillo & Cerrano, 2015). Loss of large proportions of filter feeding animals will impact ecosystem functioning and water quality and increase the risk of eutrophication and further mass mortality events. Conservation and aquaculture practices should therefore be directed at maintaining genetic diversity and monitoring the quality of the environment to reduce the synergistic effects of pollution, eutrophication, and thermal stress (Harley et al., 2006; Lejeusne et al., 2010).

## AUTHOR CONTRIBUTIONS

**Joanna Hamilton:** Conceptualization (equal); data curation (lead); formal analysis (equal); funding acquisition (equal); investigation (equal); methodology (equal); resources (supporting); software (equal); validation (equal); visualization (equal); writing – original draft (equal); writing – review and editing (lead). **Marina Piria:** Conceptualization (equal); funding acquisition (equal); methodology (equal); project administration (supporting); resources (lead); writing – review and editing (supporting). **Ana Gavrilović:** Resources (supporting); writing – review and editing (supporting). **Mirna Mrkonjić Fuka:** Resources (supporting); writing – review and editing (supporting). **Lidija Svečnjak:** Resources (supporting); writing – review and editing (supporting). **Slađana Nikolić:** Resources (supporting); writing – review and editing (supporting). **Rigers Bakiu:** Resources (supporting); writing – review and editing (supporting). **Gardner Jonathan:** Conceptualization (equal); formal analysis (equal); funding acquisition (equal); investigation (equal); methodology (equal); project administration (lead); resources (lead); software (equal); supervision (lead); validation (equal); visualization (equal); writing – original draft (equal); writing – review and editing (lead).

## Supporting information


**Data S1.** Supporting Information.

## Data Availability

The data that support the findings of this study are openly available in Dryad at [https://doi.org/10.5061/dryad.p8cz8w9t8].
