# Supplementary material for: Limited population genetic variation but pronounced seascape genetic structuring in populations of the Mediterranean mussel (Mytilus galloprovincialis) from the eastern Adriatic Sea
Source: Ecol Evol. 2023 Jan 24;13(1):e9729. doi: 10.1002/ece3.9729 (PMC9873513; doi:10.1002/ece3.9729)
Supplement: Supplementary file 1 — Data S1. Supporting Information. [file ECE3-13-e9729-s001.docx]

**Supporting Information**

Table A1. Details of microsatellite loci and amplification conditions used in this study

| Locus | Repeat Motif | Size Range (bp) | Species used for development | Source | GenBank Accession no. | Reference |
| --- | --- | --- | --- | --- | --- | --- |
| Mgµ1 | (TG)_n_ | 168 - 208 | *M. galloprovincialis* | Not stated - probably northern Spain | AF445370 | Presa et al. (2002) |
|  | 94°C, 2’; 50 × [94°C, 30”; 60°-55°C (-0.1°C/cycle), 30”, 72°C, 30”]; 72°C, 15’ | | | | | |
| MGE3 | (GTG)_n_ | 450 - 456 | *M. galloprovincialis* | EST sequences from GenBank | AJ624208 | Yu & Li (2007) |
| MGE5 | (TGA)_n_ | 220 - 244 |  |  | AJ623869 |  |
| MGE7 | (CAA)_n_ | 242 - 260 |  |  | AJ516476 |  |
|  | 94°C, 2’; 10 × [94°C, 30”; 60°-50°C (-1°C/cycle), 30”; 72°C, 30”]; 20 × [94°C, 30”; 50°C, 30”; 72°C, 30”]; 72°C, 15’ | | | | | |
| MGE4 | (CTA)_n_ | 168 - 180 |  |  | AJ624159 |  |
|  | 94°C, 2’; 40 × [94°C, 30”; 48.5°C, 30”; 72°C, 30”]; 72°C, 15’ | | | | | |
| MT203 | (CA)_n_ | 161 - 197 | *M. trossulus* | Baltic Sea | BV725482 | Gardeström et al. (2008) |
|  | 94°C, 2’; 35 × [94°C, 30”; 53°C, 30”; 72°C, 30”]; 72°C, 15’ | | | | | |
| MT282 | (GT)_n_ | 336 - 354 | *M. trossulus* | Baltic Sea | BV725484 | Gardeström et al. (2008) |
|  | 94°C, 2’; 10 × [94°C, 30”; 60°-50°C (-1°C/cycle), 30”; 72°C, 30”]; 20 × [94°C, 30”; 50°C, 30”; 72°C, 30”]; 72°C, 15’ | | | | | |

*Note*: The size range for each locus is the range given in the reference paper.

| Table A2. Data sets used in this study, showing the loci included and the number and percentage of individuals scored. | | | | | | | | | | | | | | |  |  |  |  |  |
| --- | --- | --- | --- | --- | --- | --- | --- | --- | --- | --- | --- | --- | --- | --- | --- | --- | --- | --- | --- |
| **Data set /**  Locus | **LKF** | **CK** | **KLW** | **OSOR** | **ML** | **MLC** | **Z** | **U** | **STF** | **STW** | **NF** | **BF** | **BW** | **CF** | **CW** | **IF** | **IW** | **SBF** | **Total** |
| **7 loci** | 31 | 43 | 48 | 34 | 49 | 50 | 49 | 50 | 48 | 48 | 50 | 50 | 50 | 50 | 50 | 50 | 44 | 43 | 837 |
| MGE3 | 31 | 43 | 48 | 34 | 48 | 50 | 49 | 50 | 47 | 47 | 49 | 47 | 50 | 49 | 49 | 50 | 44 | 41 | 826 |
|  | *100%* | *100%* | *100%* | *100%* | *98%* | *100%* | *100%* | *100%* | *98%* | *98%* | *98%* | *94%* | *100%* | *98%* | *98%* | *100%* | *100%* | *95%* | *99%* |
| MT282 | 29 | 34 | 43 | 28 | 38 | 46 | 36 | 42 | 45 | 43 | 45 | 44 | 46 | 34 | 39 | 42 | 37 | 41 | 712 |
|  | *94%* | *79%* | *90%* | *82%* | *78%* | *92%* | *73%* | *84%* | *94%* | *90%* | *90%* | *88%* | *92%* | *68%* | *78%* | *84%* | *84%* | *95%* | *85%* |
| MGE4 | 29 | 43 | 45 | 33 | 47 | 48 | 49 | 44 | 48 | 45 | 46 | 43 | 47 | 49 | 47 | 46 | 40 | 42 | 791 |
|  | *94%* | *100%* | *94%* | *97%* | *96%* | *96%* | *100%* | *88%* | *100%* | *94%* | *92%* | *86%* | *94%* | *98%* | *94%* | *92%* | *91%* | *98%* | *95%* |
| MGE5 | 31 | 43 | 48 | 34 | 49 | 50 | 47 | 49 | 47 | 47 | 47 | 49 | 48 | 42 | 48 | 49 | 43 | 42 | 813 |
|  | *100%* | *100%* | *100%* | *100%* | *100%* | *100%* | *96%* | *98%* | *98%* | *98%* | *94%* | *98%* | *96%* | *84%* | *96%* | *98%* | *98%* | *98%* | *97%* |
| MGE7 | 31 | 40 | 48 | 32 | 45 | 50 | 35 | 49 | 48 | 48 | 37 | 50 | 50 | 37 | 37 | 41 | 35 | 42 | 755 |
|  | *100%* | *93%* | *100%* | *94%* | *92%* | *100%* | *71%* | *98%* | *100%* | *100%* | *74%* | *100%* | *100%* | *74%* | *74%* | *82%* | *80%* | *98%* | *90%* |
| MT203 | 29 | 42 | 46 | 34 | 48 | 50 | 48 | 47 | 47 | 45 | 48 | 49 | 49 | 46 | 47 | 49 | 41 | 42 | 807 |
|  | *94%* | *98%* | *96%* | *100%* | *98%* | *100%* | *98%* | *94%* | *98%* | *94%* | *96%* | *98%* | *98%* | *92%* | *94%* | *98%* | *93%* | *98%* | *96%* |
| Mgu1 | 13 | 16 | 31 | 22 | 19 | 19 | 17 | 14 | 28 | 22 | 18 | 30 | 29 | 19 | 20 | 15 | 13 | 24 | 369 |
|  | *42%* | *37%* | *65%* | *65%* | *39%* | *38%* | *35%* | *28%* | *58%* | *46%* | *36%* | *60%* | *58%* | *38%* | *40%* | *30%* | *30%* | *56%* | *44%* |
|  |  |  |  |  |  |  |  |  |  |  |  |  |  |  |  |  |  |  |  |
| **6 loci** | 31 | 43 | 48 | 34 | 49 | 50 | 49 | 50 | 48 | 48 | 50 | 50 | 50 | 49 | 50 | 50 | 43 | 43 | 835 |
| MGE3 | 31 | 43 | 48 | 34 | 48 | 50 | 49 | 50 | 47 | 47 | 49 | 47 | 50 | 48 | 49 | 50 | 43 | 41 | 824 |
|  | *100%* | *100%* | *100%* | *100%* | *98%* | *100%* | *100%* | *100%* | *98%* | *98%* | *98%* | *94%* | *100%* | *98%* | *98%* | *100%* | *100%* | *95%* | *99%* |
| MT282 | 29 | 34 | 43 | 28 | 38 | 46 | 36 | 42 | 45 | 43 | 45 | 44 | 46 | 34 | 39 | 42 | 37 | 41 | 712 |
|  | *94%* | *79%* | *90%* | *82%* | *78%* | *92%* | *73%* | *84%* | *94%* | *90%* | *90%* | *88%* | *92%* | *69%* | *78%* | *84%* | *86%* | *95%* | *85%* |
| MGE4 | 29 | 43 | 45 | 33 | 47 | 48 | 49 | 44 | 48 | 45 | 46 | 43 | 47 | 48 | 47 | 46 | 39 | 42 | 789 |
|  | *94%* | *100%* | *94%* | *97%* | *96%* | *96%* | *100%* | *88%* | *100%* | *94%* | *92%* | *86%* | *94%* | *98%* | *94%* | *92%* | *91%* | *98%* | *94%* |
| MGE5 | 31 | 43 | 48 | 34 | 49 | 50 | 47 | 49 | 47 | 47 | 47 | 49 | 48 | 41 | 48 | 49 | 42 | 42 | 811 |
|  | *100%* | *100%* | *100%* | *100%* | *100%* | *100%* | *96%* | *98%* | *98%* | *98%* | *94%* | *98%* | *96%* | *84%* | *96%* | *98%* | *98%* | *98%* | *97%* |
| MGE7 | 31 | 40 | 48 | 32 | 45 | 50 | 35 | 49 | 48 | 48 | 37 | 50 | 50 | 37 | 37 | 41 | 35 | 42 | 755 |
|  | *100%* | *93%* | *100%* | *94%* | *92%* | *100%* | *71%* | *98%* | *100%* | *100%* | *74%* | *100%* | *100%* | *76%* | *74%* | *82%* | *81%* | *98%* | *90%* |
| MT203 | 29 | 42 | 46 | 34 | 48 | 50 | 48 | 47 | 47 | 45 | 48 | 49 | 49 | 46 | 47 | 49 | 41 | 42 | 807 |
|  | *94%* | *98%* | *96%* | *100%* | *98%* | *100%* | *98%* | *94%* | *98%* | *94%* | *96%* | *98%* | *98%* | *94%* | *94%* | *98%* | *95%* | *98%* | *97%* |
|  |  |  |  |  |  |  |  |  |  |  |  |  |  |  |  |  |  |  |  |
| **5 loci** | same as 6 loci except without MGE7 locus | | | | | |  |  |  |  |  |  |  |  |  |  |  |  |  |
|  |  |  |  |  |  |  |  |  |  |  |  |  |  |  |  |  |  |  |  |
| **Complete dataset of 577 individuals with no missing data (for *F*_ST_ calculations in seascape analyses)** | | | | | | | | | | | | |  |  |  |  |  |  |  |
| **6 loci** | 25 | 31 | 39 | 25 | 33 | 44 | 26 | 33 | 42 | 37 | 26 | 35 | 41 | 21 | 25 | 30 | 25 | 39 | 577 |
| (excl. Mgµ1) | *100%* | *100%* | *100%* | *100%* | *100%* | *100%* | *100%* | *100%* | *100%* | *100%* | *100%* | *100%* | *100%* | *100%* | *100%* | *100%* | *100%* | *100%* | *100%* |

Table A3. Data sources for environmental variables (sea surface temperature (SST), salinity (SAL) and chlorophyll-a (CHL-a)) and details of the data collection method.

| Source | Environmental variables | Method of data collection | Collection details | Sampling Site |
| --- | --- | --- | --- | --- |
| Hamer et al. (2010) | SST, SAL | Direct measurement | 3-monthly, 1998-2007 | LKF, CK, Z, STW |
| Klöppel et al. (2011) | SST, SAL | Direct measurement | May & Sept 2005 | LKF |
| Kovačić et al. (2017) | SST, SAL | Direct measurement | April & July 2010 | LKF |
| Kanduč et al. (2018) | SST, SAL | Direct measurement | Feb, Aug 2010, April, Nov 2011 | LKF |
| Ninčević-Gladan et al. (2015) | SST, SAL, CHL-a | Direct measurement | Monthly, 2001-2007 | STW, BF, BW |
| Kršinić et al. (2016) | SST, SAL, CHL-a | Direct measurement | Weekly or fortnightly 1/2002-1/2003 | NF, BF, BW |
| Mladineo et al. (2009) | SST, SAL | Direct measurement | Seasonally 2005 | NF, BF, BW |
| Hafner et al. (2018a) | SST, SAL | Direct measurement | Mar, May, Jul 2010, monthly 2011 | NF |
| Hafner et al. (2018b) | SST, SAL, CHL-a | Direct measurement | Monthly 2011 | NF |
| Gavrilović et al. (2011) | SST, SAL | Direct measurement | 2-monthly 2009 | NF, BF, BW |
| Mandić et al. (2017) | SST, SAL, CHL-a | Direct measurement | Monthly | CF, CW, IF, IW |
| Krivokapić et al. (2011) Drakulović et al. (2012) | SST, SAL, CHL-a | Direct measurement | Weekly March 2008 - Feb 2009 | IF, IW |
| Đurović et al. (2018) | CHL-a | Direct measurement | 14 times from 15/3/2015 - 28/12/15 | CF, CW, IF, IW |
| Topi et al. (2013) | SST, SAL | Direct measurement | Seasonally May 2010 - Jan 2011 | SBF |
| Kolitari et al. (2013) | SST, SAL, CHL-a | Direct measurement | Seasonally Oct 2010 - Jun 2011 | SBF |
| Moisiu et al. (2016) | SST, SAL | Direct measurement | July 2004 | SBF |
| SeaDataNet.org^1^ | SST, SAL, CHL-a | Standardised collection of ocean datasets from over 700 institutions | https://www.seadatanet.org/About-us Autumn 2011, Winter 2006 Pixels - 10 km2 | All populations except CF, CW, IF, IW, SBF |
| IOR (2022)^2^ | SST, SAL | Direct measurement | Fortnightly from mid-May to mid-Sept 2013-2017 | All populations except CF, CW, IF, IW, SBF |
| Böhm et al. (2003) | SST | Daily satellite data (NOAA-14 satellite) | Monthly median 1999-2000, Pixels - 1 km^2^ | All populations except CF, CW, IF, IW, SBF |
|  | CHL-a | Daily satellite data (SeaWiFS ocean colour) | Monthly average 1999-2000, Pixels - 1 km^2^ |  |
| Lipizer et al. (2014) | SST, SAL | DIVA modelling based on data held at NODC^3^ | Seasonal figures from Data-Interpolating Variational Analysis | All populations |
| Notes: |  |  |  |  |
| ^1^Data was originally accessed from http://sdn.oceanbrowser.net/web-vis/ and http://gher-diva.phys.ulg (28/10/18). | | | | |
| The data is now available from https://www.seadatanet.org/Products#/metadata/2a2aa0c5-4054-4a62-a18b-3835b304fe64. | | | | |
| ^2^https://vrtlac.izor.hr/ords/kakvoca/kakvoca_detalji10 (Accessed 2/10/2018) | | | |  |
| ^3^Italian Oceanographic Data Centre | |  |  |  |

References for Table A3

Böhm E, Banzon V, D’Acunzo E, D’Ortenzo F, Santoleri R (2003) Adriatic Sea surface temperature and ocean colour variability during the MFSPP. *Annales Geophysicae*, **21**, 137-149. https://doi.org/10.5194/angeo-21-137-2003

Drakulović D, Pestorić B, Cvijan M, Krivokapić S, Vuksanović N (2012) Distribution of phytoplankton community in Kotor Bay (south-eastern Adriatic Sea). *Central European Journal of Biology*. **7**(3), 470-486.  https://doi.org/10.2478/s11535-012-0023-6

Đurović B, Đurović I, Joksimović A, Crnojević V, Đukanović S, Pestorić B (2018) Monitoring the eutrophication using Landsat 8 in the Boka Kotorska Bay. *Acta Adriatica*, **59**(1), 17-34. https://doi.org/10.32582/aa.59.1.2

Gavrilović A, Jug-Dujakovic J, Marinovic Bonacic A, Conides A, Bonacic K, Ljubicic A, Van Gorder S (2011) The influence of environmental parameters on the growth and meat quality of the Mediterranean mussel *Mytilus galloprovincialis* (Mollusca: Bivalvia). *Aquaculture, Aquarium, Conservation and Legislation International Journal of the Bioflux Society*, **4**(5), 573-583.

Hafner D, Jasprica N, Car A (2018a) Taxonomic Survey of Benthic Diatoms in Neum Bay, Southeastern Adriatic. *Natura Croatica*, **27**(1), 1-26. https://doi.org/10.20302/NC.2018.27.1

Hafner D, Car A, Jasprica N, Kapetanović T, Dupčić Radić I (2018b) Relationship between marine epilithic diatoms and environmental variables in oligotrophic bay, NE Mediterranean. *Mediterranean Marine Science*, **19**(2), 223-239. https://doi.org/10.12681/mms.14151

Hamer B, Medaković D, Pavičić-Hamer D, Jakšić Ž, Štifanić M, Nerlović V, Travizi1 A, Precali R, Kanduč T (2010) Estimation of freshwater influx along the eastern Adriatic coast as a possible source of stress for marine organisms. *Acta Adriatica*, **51**(2), 181-194. UDC: 551.465.8 : 594.124 (262.3-11)

Kanduč T, Šlejkovec Z, Falnogaa I, Mori N, Budič B, Kovačić I, Pavičić-Hamer D, Hamer B (2018) Environmental status of the NE Adriatic Sea, Istria, Croatia: Insights from mussel Mytilus galloprovincialis condition indices, stable isotopes and metal(loid)s. *Marine Pollution Bulletin*, **126**, 525-534. https://doi.org/10.1016/j.marpolbul.2017.09.052

Klöppel A, Messal C, Pfannkuchen M, Matschullat J, Zucht W, Hamer B, Brümmer F (2011) Abiotic Sponge Ecology Conditions, Limski Kanal and Northern Adriatic Sea, Croatia. *Open Journal of Marine Science*, **1**, 18-30. https://doi.org/10.4236/ojms.2011.11002

Kolitari J, Gjyli L, Mukli L, Gjyli S, Vukaj J (2013) Distribution of Chlorophyll a in Lagoon of Butrint waters comparing with environment factors (Albania)*. Albanian Journal of Agricultural Sciences*, **12**(1), 87-9.

Kovačić I, Pavičić-Hamer D, Kanduč T, Hamer B (2017) Adaptation of cultured mussel *Mytilus galloprovincialis* Lamarck, 1819 from the northern Adriatic Sea to nearby aquaculture sites and translocation. *Acta Adriatica*, **58**(2), 285-296. https://doi.org/10.32582/aa.58.2.8

Krivokapić S, Pestorić B, Bosak S, Kušpilić G, Wexels Riser C (2011) Trophic State of Boka Kotorska Bay (South-eastern Adriatic Sea). *Fresenius Environmental Bulletin*, **20**(8), 1960-1969.

Kršinić F, Čalić M, Carić M (2016) The population structure of planktonic protists and small metazoans in Mali Ston Bay (Adriatic Sea) - implications for determination of trophic state and shellfish culturing potential. *Acta Adriatica*, **57**(1), 17-38.

Lipizer M, Partescano E, Rabitti A, Giorgetti A, Crise A (2014) Qualified temperature, salinity and dissolved oxygen climatologies in a changing Adriatic Sea. *Ocean Science*, **10**, 771-797. https://doi.org/10.5194/os-10-771-2014

Mandić M, Ikica Z, Gvozdenović S (2017) Mariculture in the Boka Kotorska Bay: Tradition, Current State and Perspective. In: *The Boka Kotorska Bay Environment* (eds Joksimović A, Djurović M, Semenov AV, Zonn IS, Kostianoy AG), pp. 395-409. Springer, Cham. https://doi.org/10.1007/698_2016_33

Mladineo I, Trumbić Ž, Jozić S, Šegvić T (2009) First Report of Cryptosporidium sp. (Coccidia, Apicomplexa) Oocysts in the Black Mussel (*Mytilus galloprovincialis*) reared in the Mali Ston Bay, Adriatic Sea, *Journal of Shellfish Research*, **28**(3), 541-543. https://doi.org/10.2983/035.028.0316

Moisiu L, Panagiotopoulos IP, Durmishi Ҫ, Kapsimalis V, Anagnostou C (2016) The anoxic Butrint Lagoon, SW Albania. *Environmental Earth Sciences*, **75**, 1443. https://doi.org/10.1007/s12665-016-6259-0

Ninčević-Gladan Z, Bužančić M, Kušpilić G, Grbec B, Matijević S, Skejić S, Marasović I, Morović M (2015) The response of phytoplankton community to anthropogenic pressure gradient in the coastal waters of the eastern Adriatic Sea. *Ecological Indicators*, **56**, 106-115. https://doi.org/10.1016/j.ecolind.2015.03.018

Topi T, Bani A, Sulçe S (2013) Physico chemical characteristics and heavy metal contents of water from Butrinti lagoon, Albania. *Albanian Journal of Agricultural Sciences*, **12**(2), 321-326.

Table A4. The environmental and geospatial variables used in the seascape analyses.

|  | min SST | max SST | delta SST | min SAL | max SAL | delta SAL | min CHL-a | max CHL-a | delta CHL-a | lat | long | Total CD |
| --- | --- | --- | --- | --- | --- | --- | --- | --- | --- | --- | --- | --- |
| LKF | 8.2 | 27.2 | 19.0 | 34.6 | 37.5 | 2.9 | 0.3 | 0.8 | 0.5 | 45.13334 | 13.66667 | 5975 |
| CK | 9.4 | 27.2 | 17.8 | 32.2 | 36.5 | 4.3 | 0.2 | 0.7 | 0.5 | 45.24935 | 14.58243 | 5487 |
| KLW | 9.4 | 28.0 | 18.6 | 33.4 | 36.4 | 3.0 | 3.0 | 3.0 | 0 | 45.15497 | 14.61889 | 5657 |
| OSOR | 9.0 | 26.0 | 17.0 | 36.7 | 37.3 | 0.6 | 0.1 | 1.0 | 0.9 | 44.69283 | 14.39231 | 4701 |
| ML | 9.0 | 26.0 | 17.0 | 36.7 | 37.6 | 0.9 | 0.2 | 0.6 | 0.4 | 44.53228 | 14.46835 | 4609 |
| MLC | 9.0 | 26.0 | 17.0 | 36.7 | 37.6 | 0.9 | 0.2 | 0.6 | 0.4 | 44.53181 | 14.45143 | 4474 |
| Z | 10.0 | 26.0 | 16.0 | 33.7 | 38.3 | 4.6 | 0.4 | 2.0 | 1.6 | 44.09785 | 15.25289 | 3882 |
| U | 10.0 | 25.6 | 15.6 | 34.3 | 37.9 | 3.6 | 0.2 | 1.0 | 0.8 | 44.01455 | 15.25086 | 3837 |
| STF | 10.0 | 27.1 | 17.1 | 35.4 | 37.5 | 2.1 | 0.3 | 1.2 | 0.9 | 43.51543 | 16.13917 | 4010 |
| STW | 10.0 | 28.3 | 18.3 | 30.1 | 37.5 | 7.4 | 0.4 | 2.5 | 2.1 | 43.53180 | 16.46667 | 4025 |
| NF | 10.0 | 28.5 | 18.5 | 28.4 | 38.0 | 9.6 | 0.3 | 1.2 | 0.9 | 42.91312 | 17.62381 | 4455 |
| BF | 6.9 | 28.5 | 21.6 | 29.1 | 38.6 | 9.5 | 0.2 | 1.8 | 1.6 | 42.87976 | 17.70917 | 4825 |
| BW | 6.9 | 28.5 | 21.6 | 29.1 | 38.6 | 9.5 | 0.2 | 1.8 | 1.6 | 42.86880 | 17.69835 | 4660 |
| CF | 7.0 | 31.4 | 24.4 | 9.2 | 35.2 | 26.0 | 0.1 | 7.0 | 6.9 | 42.48539 | 18.74596 | 5445 |
| CW | 7.0 | 31.4 | 24.4 | 9.2 | 35.2 | 26.0 | 0.1 | 7.0 | 6.9 | 42.48568 | 18.74349 | 5445 |
| IF | 7.0 | 31.4 | 24.4 | 9.2 | 35.2 | 26.0 | 0.1 | 7.0 | 6.9 | 42.43705 | 18.76321 | 5515 |
| IW | 7.0 | 31.4 | 24.4 | 9.2 | 35.2 | 26.0 | 0.1 | 7.0 | 6.9 | 42.43596 | 18.76367 | 5515 |
| SBF | 10.1 | 28.2 | 18.1 | 13.0 | 32.8 | 19.8 | 0.1 | 1.9 | 1.8 | 39.75474 | 20.03251 | 9865 |

Table A5. The 7 best-fitting models for the genetic index frequency of allele MGE7^243^, also showing the percentage of best-fitting models including each environmental variable.

| Model Rank | minSST | minSAL | maxSAL | minCHL-a | Lat | Total CD |
| --- | --- | --- | --- | --- | --- | --- |
| 1 | - | ✓ | - | - | - | - |
| 2 | - | ✓ | - | - | ✓ | - |
| 3 | ✓ | ✓ | - | - | - | - |
| 4 | - | ✓ | - | ✓ | - | - |
| 5 | - | ✓ | ✓ | - | - | - |
| 6 | ✓ | ✓ | ✓ | - | - | - |
| 7 | - | ✓ | - | - | - | ✓ |
| All best-fit models | 28.6% | 100.0% | 28.6% | 14.3% | 14.3% | 14.3% |

Table A6. Estimates of effective population size, *Ne*, (*Ne*Estimator) with the 95% confidence intervals. *P*crit is the critical value for allele frequencies below which rare alleles were excluded from the calculation.

|  |  |  | Dataset including MGE7 | | |  | Dataset excluding MGE7 | | |
| --- | --- | --- | --- | --- | --- | --- | --- | --- | --- |
|  |  |  |  | 95% Confidence Interval | |  |  | 95% Confidence Interval | |
| Population | *P*crit |  | Ne | Lower | Upper |  | Ne | Lower | Upper |
| LKF | 0.02 |  | 46.2 | 20.4 | 618.3 |  | 42.9 | 17.3 | ∞ |
| CK | 0.02 |  | ∞ | 386.8 | ∞ |  | ∞ | 202.2 | ∞ |
| KLW | 0.02 |  | ∞ | 444.5 | ∞ |  | ∞ | 195.5 | ∞ |
| OSOR | 0.02 |  | ∞ | 190.1 | ∞ |  | ∞ | 675.4 | ∞ |
| ML | 0.02 |  | 120.6 | 49.0 | 3465.5 |  | 394.7 | 65.1 | ∞ |
| MLC | 0.02 |  | ∞ | ∞ | ∞ |  | ∞ | ∞ | ∞ |
| Z | 0.02 |  | 108.8 | 44.8 | ∞ |  | 82.3 | 36.9 | 1301.4 |
| U | 0.02 |  | 148.5 | 50.2 | ∞ |  | 189.0 | 49.3 | ∞ |
| STF | 0.02 |  | ∞ | 115.9 | ∞ |  | ∞ | 125.7 | ∞ |
| STW | 0.02 |  | 210.1 | 59.3 | ∞ |  | 224.0 | 52.2 | ∞ |
| NF | 0.02 |  | ∞ | 220.6 | ∞ |  | ∞ | 314.4 | ∞ |
| BF | 0.02 |  | ∞ | 360.4 | ∞ |  | ∞ | 138.2 | ∞ |
| BW | 0.02 |  | 157.9 | 60.9 | ∞ |  | 237.8 | 62.1 | ∞ |
| CF | 0.025 |  | 88.8 | 35.8 | ∞ |  | 384.8 | 53.9 | ∞ |
| CW | 0.02 |  | 346.4 | 69.4 | ∞ |  | 198.9 | 55.5 | ∞ |
| IF | 0.02 |  | ∞ | 252.6 | ∞ |  | ∞ | 150.7 | ∞ |
| IW | 0.02 |  | 76.4 | 35.6 | 774.6 |  | 50.0 | 26.2 | 161.1 |
| SBF | 0.02 |  | ∞ | 108.4 | ∞ |  | ∞ | ∞ | ∞ |

Table A7. Measures of genetic diversity for 18 populations of *Mytilus galloprovincialis*: A_R_ allelic richness, PA_R_ private allelic richness, P_a_ number of private alleles, H_O_ observed heterozygosity, H_E_ expected heterozygosity, *F*_IS_ inbreeding coefficient.

| Population | A_R_ | PA_R_ | P_a_ | H_O_ | H_E_ | *F*_IS_ |
| --- | --- | --- | --- | --- | --- | --- |
| LKF | 6.56 | 0.12 | 0 | 0.399 | 0.629 | 0.335 |
| CK | 7.90 | 0.05 | 0 | 0.427 | 0.659 | 0.345 |
| KLW | 6.95 | 0.10 | 1 | 0.360 | 0.628 | 0.404 |
| OSOR | 7.06 | 0.01 | 0 | 0.422 | 0.659 | 0.321 |
| ML | 6.76 | 0.01 | 0 | 0.422 | 0.606 | 0.267 |
| MLC | 7.35 | 0.11 | 1 | 0.409 | 0.662 | 0.365 |
| Z | 6.76 | 0.02 | 0 | 0.381 | 0.638 | 0.379 |
| U | 6.51 | 0.22 | 2 | 0.403 | 0.629 | 0.333 |
| STF | 7.52 | 0.40 | 3 | 0.380 | 0.663 | 0.410 |
| STW | 7.05 | 0.12 | 1 | 0.413 | 0.636 | 0.334 |
| NF | 6.16 | 0.10 | 1 | 0.323 | 0.558 | 0.402 |
| BF | 6.94 | 0.11 | 1 | 0.424 | 0.651 | 0.340 |
| BW | 6.89 | 0.10 | 1 | 0.436 | 0.656 | 0.312 |
| CF | 6.60 | 0.01 | 0 | 0.329 | 0.567 | 0.419 |
| CW | 6.75 | 0.33 | 2 | 0.358 | 0.579 | 0.368 |
| IF | 6.98 | 0.15 | 1 | 0.372 | 0.640 | 0.404 |
| IW | 7.12 | 0.15 | 1 | 0.336 | 0.627 | 0.453 |
| SBF | 7.03 | 0.00 | 0 | 0.404 | 0.641 | 0.376 |

| Table A8. Pairwise *F*_ST_ values calculated by AMOVA for 18 populations of *Mytilus galloprovincialis* based on all 6 loci below the diagonal and on 5 neutral loci above. *F*_ST_ values with significant p-values (after FDR correction) in bold. |
| --- |
| \|  \| LKF \| CK \| KLW \| OSOR \| ML \| MLC \| Z \| U \| STF \| STW \| NF \| BF \| BW \| CF \| CW \| IF \| IW \| SBF \| Mean F_ST_ \| \| --- \| --- \| --- \| --- \| --- \| --- \| --- \| --- \| --- \| --- \| --- \| --- \| --- \| --- \| --- \| --- \| --- \| --- \| --- \| --- \| \| LKF \|  \| 0.001 \| 0.000 \| 0.004 \| 0.015 \| 0.000 \| 0.008 \| 0.000 \| 0.000 \| 0.000 \| 0.000 \| 0.000 \| 0.000 \| 0.004 \| 0.003 \| 0.000 \| 0.000 \| 0.000 \| 0.002 \| \| CK \| 0.010 \|  \| 0.003 \| 0.000 \| 0.009 \| 0.002 \| 0.010 \| 0.007 \| 0.001 \| 0.000 \| 0.004 \| 0.000 \| 0.001 \| 0.000 \| 0.000 \| 0.000 \| 0.000 \| 0.001 \| 0.002 \| \| KLW \| 0.011 \| 0.003 \|  \| 0.000 \| 0.006 \| 0.000 \| 0.010 \| 0.014 \| 0.002 \| 0.000 \| 0.007 \| 0.002 \| 0.000 \| 0.001 \| 0.005 \| 0.001 \| 0.006 \| 0.000 \| 0.003 \| \| OSOR \| **0.015** \| 0.000 \| 0.000 \|  \| 0.000 \| 0.000 \| 0.004 \| 0.004 \| 0.000 \| 0.000 \| 0.008 \| 0.004 \| 0.000 \| 0.000 \| 0.000 \| 0.000 \| 0.000 \| 0.002 \| 0.002 \| \| ML \| **0.016** \| 0.007 \| **0.010** \| 0.000 \|  \| 0.009 \| 0.005 \| 0.012 \| 0.009 \| 0.009 \| 0.014 \| 0.016 \| 0.008 \| 0.001 \| 0.007 \| 0.010 \| 0.009 \| 0.014 \| 0.009 \| \| MLC \| 0.000 \| 0.001 \| 0.000 \| 0.000 \| 0.007 \|  \| 0.004 \| 0.005 \| 0.000 \| 0.000 \| 0.005 \| 0.000 \| 0.000 \| 0.004 \| 0.004 \| 0.000 \| 0.000 \| 0.000 \| 0.002 \| \| Z \| **0.029** \| **0.012** \| **0.019** \| 0.008 \| **0.011** \| **0.015** \|  \| 0.007 \| 0.014 \| 0.011 \| **0.017** \| 0.009 \| 0.002 \| 0.001 \| 0.011 \| 0.005 \| 0.010 \| 0.014 \| 0.008 \| \| U \| 0.006 \| 0.003 \| **0.011** \| 0.002 \| **0.009** \| 0.003 \| **0.013** \|  \| 0.003 \| 0.000 \| 0.000 \| 0.002 \| 0.002 \| 0.007 \| 0.000 \| 0.000 \| 0.003 \| 0.009 \| 0.004 \| \| STF \| 0.000 \| 0.001 \| 0.005 \| 0.002 \| 0.007 \| 0.000 \| **0.025** \| 0.004 \|  \| 0.000 \| 0.000 \| 0.000 \| 0.000 \| 0.002 \| 0.000 \| 0.001 \| 0.000 \| 0.000 \| 0.002 \| \| STW \| 0.006 \| 0.000 \| 0.000 \| 0.000 \| 0.005 \| 0.000 \| **0.017** \| 0.000 \| 0.000 \|  \| 0.000 \| 0.000 \| 0.000 \| 0.000 \| 0.000 \| 0.000 \| 0.000 \| 0.000 \| 0.001 \| \| NF \| **0.019** \| 0.006 \| **0.016** \| **0.011** \| **0.016** \| **0.015** \| **0.011** \| 0.007 \| **0.014** \| **0.009** \|  \| 0.002 \| 0.003 \| 0.002 \| 0.000 \| 0.001 \| 0.000 \| 0.007 \| 0.004 \| \| BF \| 0.000 \| 0.000 \| 0.005 \| 0.005 \| **0.013** \| 0.000 \| **0.020** \| 0.001 \| 0.000 \| 0.000 \| **0.013** \|  \| 0.000 \| 0.000 \| 0.001 \| 0.000 \| 0.000 \| 0.000 \| 0.002 \| \| BW \| 0.000 \| 0.001 \| 0.000 \| 0.003 \| 0.006 \| 0.000 \| **0.015** \| 0.001 \| 0.000 \| 0.000 \| **0.012** \| 0.000 \|  \| 0.000 \| 0.000 \| 0.000 \| 0.000 \| 0.000 \| 0.001 \| \| CF \| **0.028** \| **0.011** \| **0.030** \| **0.017** \| 0.009 \| **0.025** \| **0.011** \| **0.022** \| **0.023** \| **0.014** \| **0.013** \| **0.017** \| **0.016** \|  \| 0.000 \| 0.000 \| 0.000 \| 0.002 \| 0.001 \| \| CW \| **0.023** \| 0.004 \| **0.026** \| **0.013** \| **0.011** \| **0.019** \| **0.015** \| **0.012** \| **0.014** \| 0.007 \| 0.002 \| **0.014** \| **0.011** \| 0.000 \|  \| 0.000 \| 0.000 \| 0.003 \| 0.002 \| \| IF \| **0.016** \| 0.000 \| 0.007 \| 0.000 \| **0.012** \| 0.004 \| 0.004 \| 0.000 \| **0.010** \| 0.001 \| 0.000 \| 0.003 \| 0.004 \| **0.009** \| 0.000 \|  \| 0.000 \| 0.003 \| 0.001 \| \| IW \| **0.014** \| 0.000 \| **0.009** \| 0.001 \| **0.009** \| 0.005 \| 0.006 \| 0.005 \| 0.008 \| 0.000 \| 0.000 \| 0.006 \| 0.005 \| **0.009** \| 0.002 \| 0.000 \|  \| 0.005 \| 0.002 \| \| SBF \| 0.008 \| 0.005 \| 0.008 \| 0.010 \| **0.012** \| 0.001 \| **0.027** \| **0.013** \| 0.003 \| 0.000 \| **0.019** \| 0.001 \| 0.000 \| **0.015** \| **0.009** \| **0.011** \| **0.010** \|  \| 0.004 \| \| Mean F_ST_ \| 0.012 \| 0.004 \| 0.009 \| 0.005 \| 0.009 \| 0.006 \| 0.015 \| 0.007 \| 0.007 \| 0.003 \| 0.011 \| 0.006 \| 0.004 \| 0.016 \| 0.011 \| 0.005 \| 0.005 \| 0.009 \|  \| |

Table A9. Correlation coefficients, *R*, between nine environmental and three geospatial variables. After the removal of highly correlated variables, |R| > 0.85, the final data set consisted of 6 variables - minSAL, minSST, maxSAL, minCHL-a, Lat and TotalCD.

|  | minSST | maxSST | deltaSST | minSAL | maxSAL | deltaSAL | minCHL-a | maxCHL-a | deltaCHL-a | Lat | Long | Total CD |
| --- | --- | --- | --- | --- | --- | --- | --- | --- | --- | --- | --- | --- |
| minSST | 1.000 |  |  |  |  |  |  |  |  |  |  |  |
| maxSST | -0.704 | 1.000 |  |  |  |  |  |  |  |  |  |  |
| deltaSST | -0.886 | 0.953 | 1.000 |  |  |  |  |  |  |  |  |  |
| minSAL | 0.571 | -0.911 | -0.838 | 1.000 |  |  |  |  |  |  |  |  |
| maxSAL | 0.154 | -0.574 | -0.440 | 0.783 | 1.000 |  |  |  |  |  |  |  |
| deltaSAL | -0.614 | 0.928 | 0.868 | -0.995 | -0.718 | 1.000 |  |  |  |  |  |  |
| minCHL-a | 0.221 | -0.090 | -0.153 | 0.242 | 0.028 | -0.266 | 1.000 |  |  |  |  |  |
| maxCHL-a | -0.654 | 0.918 | 0.878 | -0.891 | -0.592 | 0.904 | -0.044 | 1.000 |  |  |  |  |
| deltaCHL-a | -0.682 | 0.900 | 0.878 | -0.913 | -0.573 | 0.931 | -0.299 | 0.966 | 1.000 |  |  |  |
| Lat | 0.237 | -0.576 | -0.477 | 0.757 | 0.618 | -0.749 | 0.359 | -0.462 | -0.534 | 1.000 |  |  |
| Long | -0.445 | 0.794 | 0.708 | -0.874 | -0.594 | 0.883 | -0.301 | 0.672 | 0.719 | -0.940 | 1.000 |  |
| Total CD | -0.056 | 0.287 | 0.211 | -0.505 | -0.813 | 0.436 | 0.022 | 0.175 | 0.161 | -0.586 | 0.455 | 1.000 |


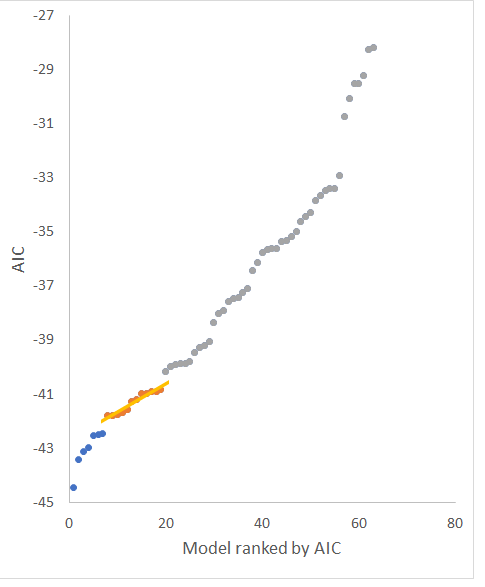


Figure A1. Plot of successive AIC values (smallest to largest), in this case for the frequency of allele MGE7^243^, f(MGE7^243^). The group of best-fitting models reported included all models below the point of inflexion of the AIC plot (the orange line on the plot), here: models 1 – 7. The top-ranked model included minSAL alone and all 7 of the group of best fitting models included the variable minSAL.

| 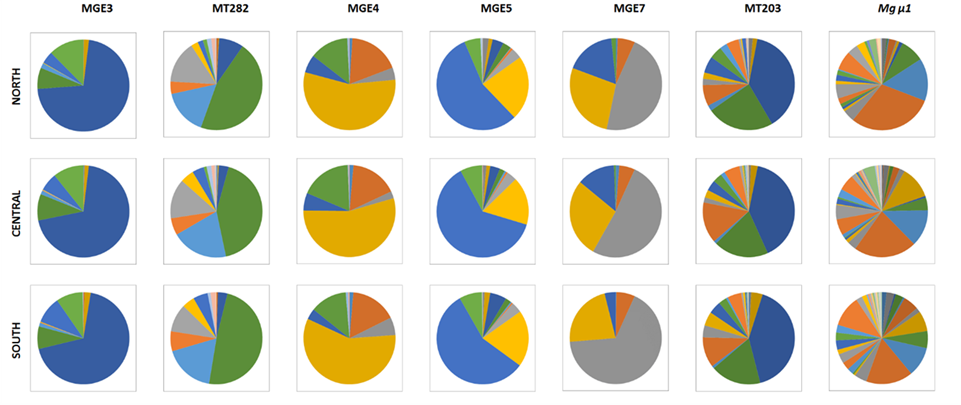 |
| --- |

Figure A2. Allelic frequencies for 7 loci for each of the three regions.

| (a) 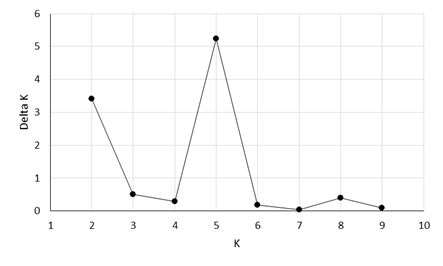 | (b) 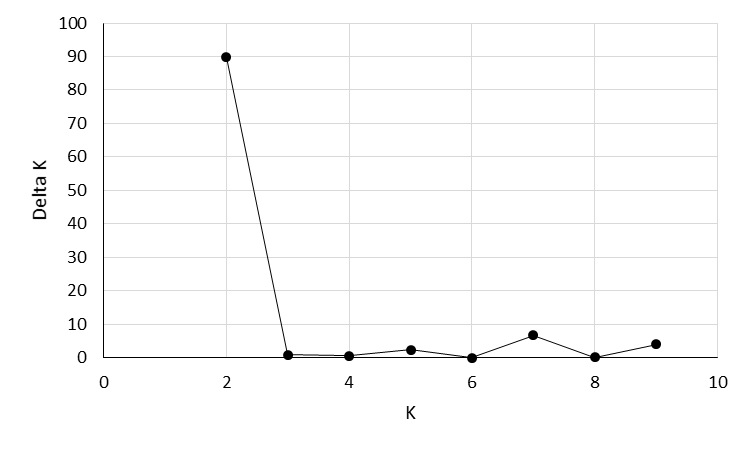 |
| --- | --- |
| (c) 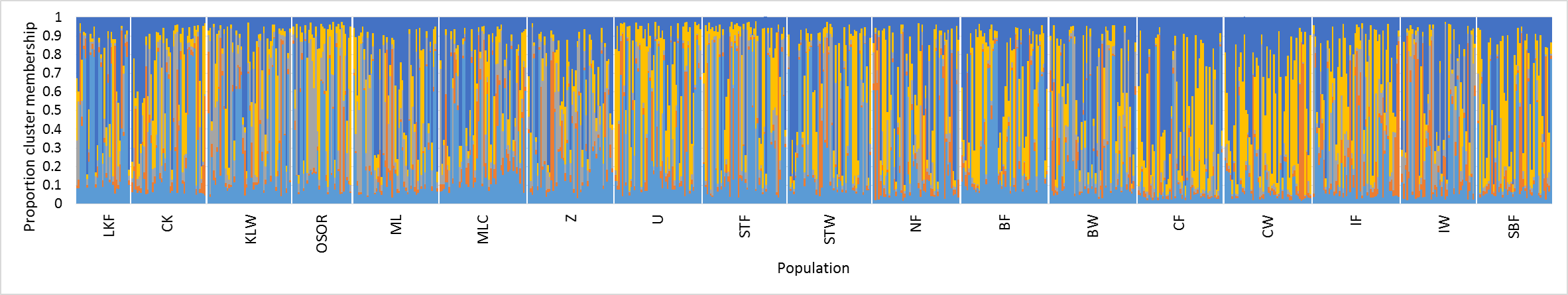 | |
| (d) 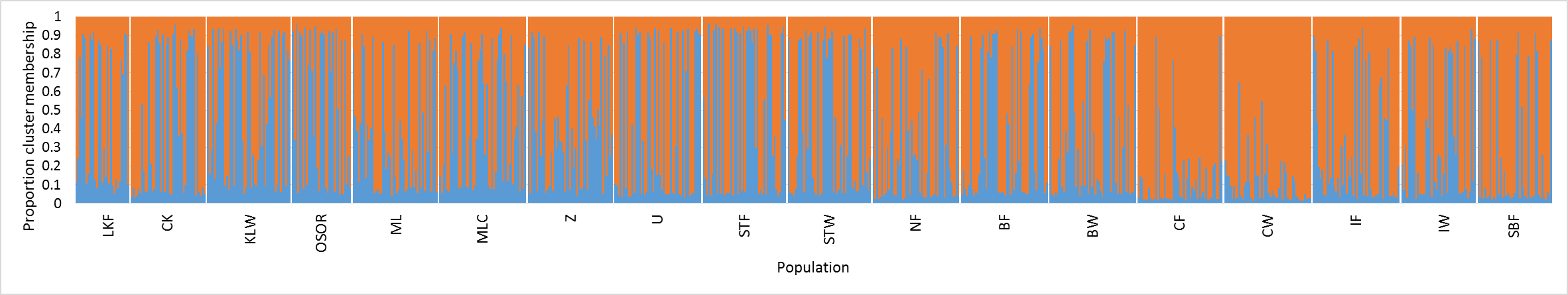 | |
| (e) 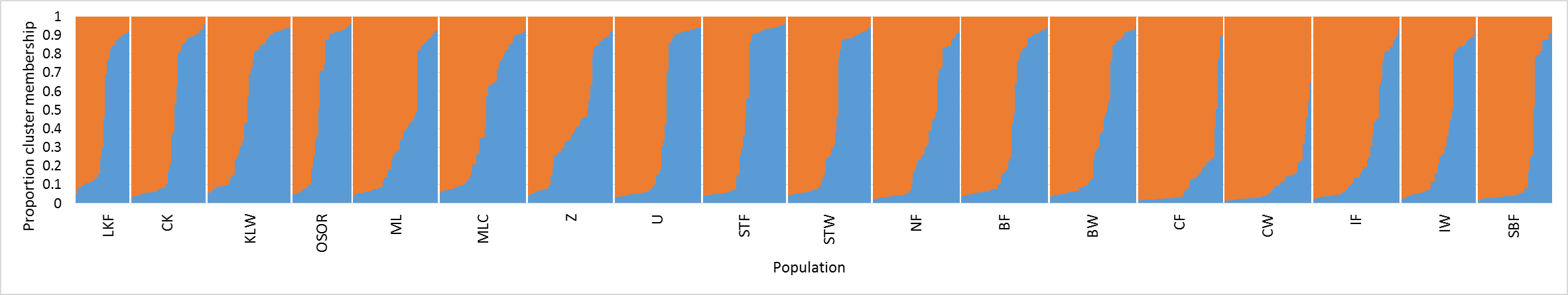 | |

Figure A3. Structure analysis results for *Mytilus galloprovincialis* using 6 loci: Structure Harvester results using (a) the Admixture model with a maximum at *K* = 5 and local maximum at *K* = 2, and (b) the No admixture model with maximum at *K* = 2; bar plots of structure outputs using the Admixture model for (c) *K* = 5 and (d) *K* = 2 and (e) *K* = 2 with individuals sorted by proportion cluster membership within population

| (a) | (b) |
| --- | --- |
| 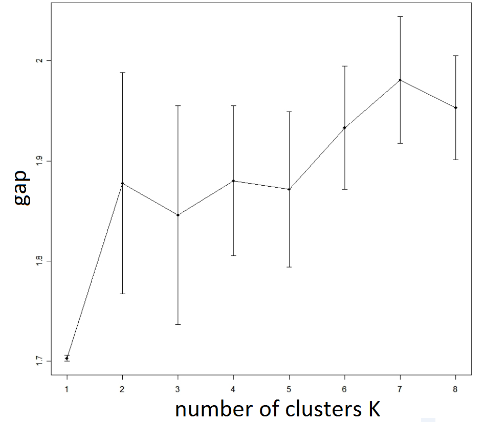 | 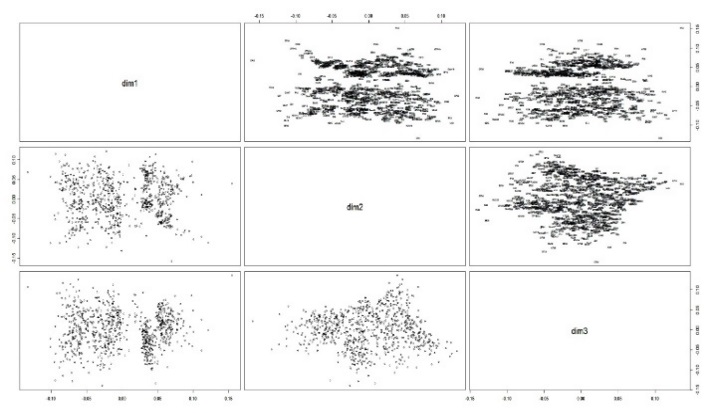 |
| (c) | (d) |
| 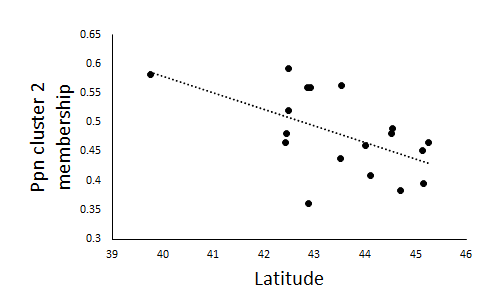 | 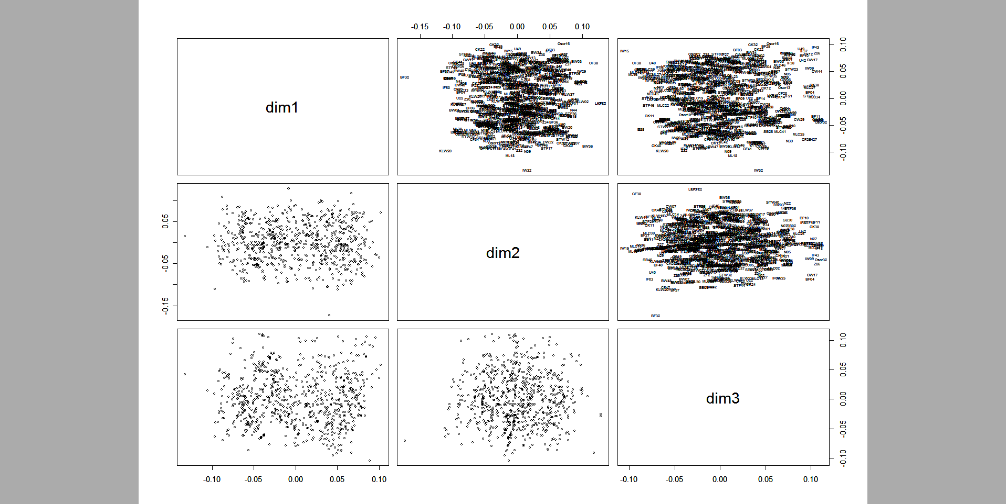 |

Figure A4. AWclust results for *Mytilus galloprovincialis*: (a) the gap statistic showing the optimal number of clusters, (b) 2-D MDS cluster plot of the allele sharing distance, (c) Linear regression analysis of proportion of cluster 2 membership as a function of latitude, R^2^ = 0.308, p = 0.017, and using 5 neutral loci: (d) 2-D MDS cluster plot of the allele sharing distance.

| (a)  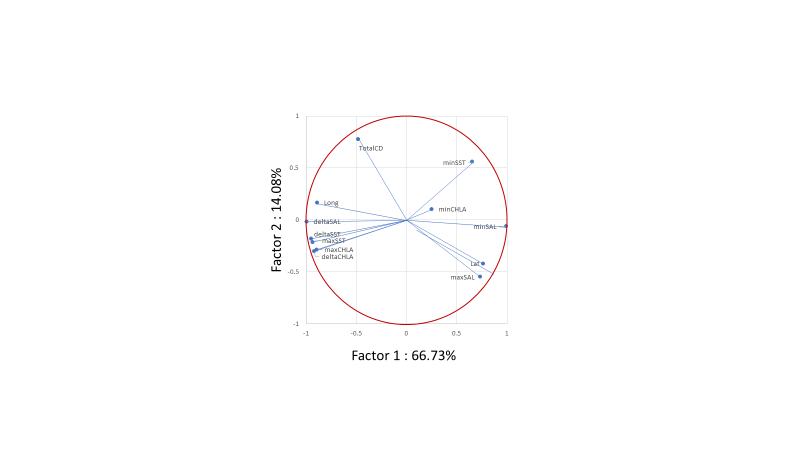 | (b)  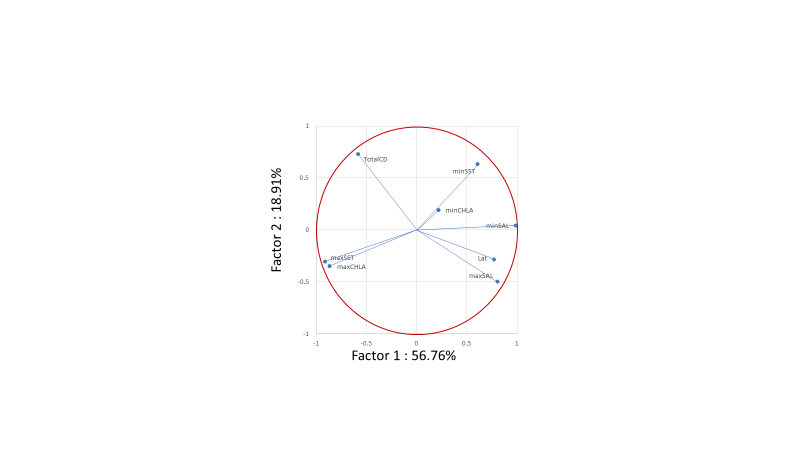 |
| --- | --- |

Figure A5. Principal component analysis (PCA) plots of (a) 12 environmental and geospatial variables and (b) 8 variables after removal of 4 highly correlated variables.
